# Correction: Tran et al. Antimicrobial Properties of *Bacillus* Probiotics as Animal Growth Promoters. *Antibiotics* 2023, *12*, 407

**DOI:** 10.3390/antibiotics12091420

**Published:** 2023-09-08

**Authors:** Charlie Tran, Darwin Horyanto, Dragana Stanley, Ian E. Cock, Xiaojing Chen, Yunjiang Feng

**Affiliations:** 1 Griffith Institute for Drug Discovery (GRIDD), Griffith University, Brisbane, QLD 4111, Australia; 2 Institute for Future Farming Systems, Central Queensland University, Rockhampton, QLD 4702, Australia; 3 Bioproton Pty Ltd., Brisbane, QLD 4110, Australia; 4 School of Environment and Science, Griffith University, Brisbane, QLD 4111, Australia

**Error in Conflicts of Interest**

In the original publication [1], **Conflicts of Interest** was described inaccurately. Please replace it with the following paragraph under the heading of **Conflicts of Interest**:

“The publication presents the results of an industry collaborative research project. The project involves students and staff from Griffith University (C.T., I.C., Y.F.), Central Queensland University (D.H., D.S.) and Bioproton Pty Ltd. (D.H., X.C.). C.T. is supported by a PhD scholarship from Bioproton Pty Ltd.” 

The authors state that the scientific conclusions are unaffected. This correction was approved by the Academic Editor. The original publication has also been updated.
