# Supplementary material for: Antimicrobial Properties of Bacillus Probiotics as Animal Growth Promoters
Source: Antibiotics (Basel). 2023 Feb 17;12(2):407. doi: 10.3390/antibiotics12020407 (PMC9952206; doi:10.3390/antibiotics12020407)

Supplementary information for

Antimicrobial Bacillus probiotics as animal growth promoters

Charlie Tran[1], Darwin Horyanto[2,4], Dragana Stanley[2], Ian E. Cock [3], Xiaojing Chen[4] and Yunjiang Feng [1,*]

[1] Griffith Institute for Drug Discovery (GRIDD), Griffith University, Nathan, QLD, 4111, Australia

[2] Institute for Future Farming Systems, Central Queensland University, Rockhampton, QLD, 4702, Australia.

[3] School of Environment and Science, Griffith University, Nathan, QLD 4111, Australia

[4] Bioproton Pty Ltd, Acacia Ridge, QLD, 4110, Australia

* Correspondence: y.feng@griffith.edu.au

List of supporting information:

Table S1 Experimental NMR data of compound 1 in DMSO - d_6 at 25°C

Figure S1 (+)-LRESIMS spectrum of compound 1

Figure S2 ^{1}H NMR (800 MHz, DMSO) spectrum of compound 1

Figure S3 ^{1}H – ^{1}H COSY spectrum of compound 1 in DMSO

Figure S4 HSQC spectrum of compound 1 in DMSO

Figure S5 HMBC spectrum of compound 1 in DMSO

Table S2 Experimental NMR data of compound 2 in DMSO - d_6 at 25°C

Figure S6 (+)-LRESIMS spectrum of compound 2

Figure S7 ^{1}H NMR (800 MHz, DMSO) spectrum of compound 2

Figure S8 ^{1}H – ^{1}H COSY spectrum of compound 2 in DMSO

Figure S9 HSQC spectrum of compound 2 in DMSO

Figure S10 HMBC spectrum of compound 2 in DMSO

Table S3 Experimental NMR data of compound 3 in DMSO - d_6 at 25°C

Figure S11 (+)-LRESIMS spectrum of compound 3

Figure S12 ^{1}H NMR (800 MHz, DMSO) spectrum of compound 3

Figure S13 $^{1}H-^{1}H$ COSY spectrum of compound 3 in DMSO

Figure S14 HSQC spectrum of compound 3 in DMSO

Figure S15 HMBC spectrum of compound 3 in DMSO

Table S4 Experimental NMR data of compound 4 in DMSO - d_6 at 25°C

Figure S16 (+)-LRESIMS spectrum of compound 4

Figure S17 ^{1}H NMR (800 MHz, DMSO) spectrum of compound 4

Figure S18 $^{1}H-^{1}H$ COSY spectrum of compound 4 in DMSO

Figure S19 HSQC spectrum of compound 4 in DMSO

Figure S20 HMBC spectrum of compound 4 in DMSO

Table S5 Experimental NMR data of compound 5 in DMSO - d_6 at 25°C

Figure S21 (+)-LRESIMS spectrum of compound 5

Figure S22 ^{1}H NMR (800 MHz, DMSO) spectrum of compound 5

Figure S23 $^{1}H-^{1}H$ COSY spectrum of compound 5 in DMSO

Figure S24 HSQC spectrum of compound 5 in DMSO

Figure S25 HMBC spectrum of compound 5 in DMSO

Table S6 Experimental NMR data of compound 6 in DMSO - d_6 at 25°C

Figure S26 (+)-LRESIMS spectrum of compound 6

Figure S27 ^{1}H NMR (800 MHz, DMSO) spectrum of compound 6

Figure S28 $^{1}H-^{1}H$ COSY spectrum of compound 6 in DMSO

Figure S29 HSQC spectrum of compound 6 in DMSO

Figure S30 HMBC spectrum of compound 6 in DMSO

Table S7 Experimental NMR data of compound 7 in DMSO - d_6 at 25°C

Figure S31 (+)-LRESIMS spectrum of compound 7

Figure S32 ^{1}H NMR (800 MHz, DMSO) spectrum of compound 7

Figure S33 $^{1}H-^{1}H$ COSY spectrum of compound 7 in DMSO

Figure S34 HSQC spectrum of compound 7 in DMSO

Figure S35 HMBC spectrum of compound 7 in DMSO

Table S8 Experimental NMR data of compound 8 in DMSO - *d*6 at 25°C

Figure S36 (+)-LRESIMS spectrum of compound 8

Figure S37 ^{1}H NMR (800 MHz, DMSO) spectrum of compound 8

Figure S38 ^{1}H – ^{1}H COSY spectrum of compound 8 in DMSO

Figure S39 HSQC spectrum of compound 8 in DMSO

Figure S40 HMBC spectrum of compound 8 in DMSO

Figure S41 LC-MS chromatograms of caecum extracts produced from a single animal sample fed with Bacillus composition F1.

Table S9 Antimicrobial activity of EtoAc and crude extracts of *Bacillus* strains (green tick: active; red cross: inactive)

Figure S42 Stacked ^{1}H NMR spectra of *Bacillus* EtoAC extracts

Table S1 Experimental NMR data of C16 Surfactin C in DMSO - *d*6 at 25°C

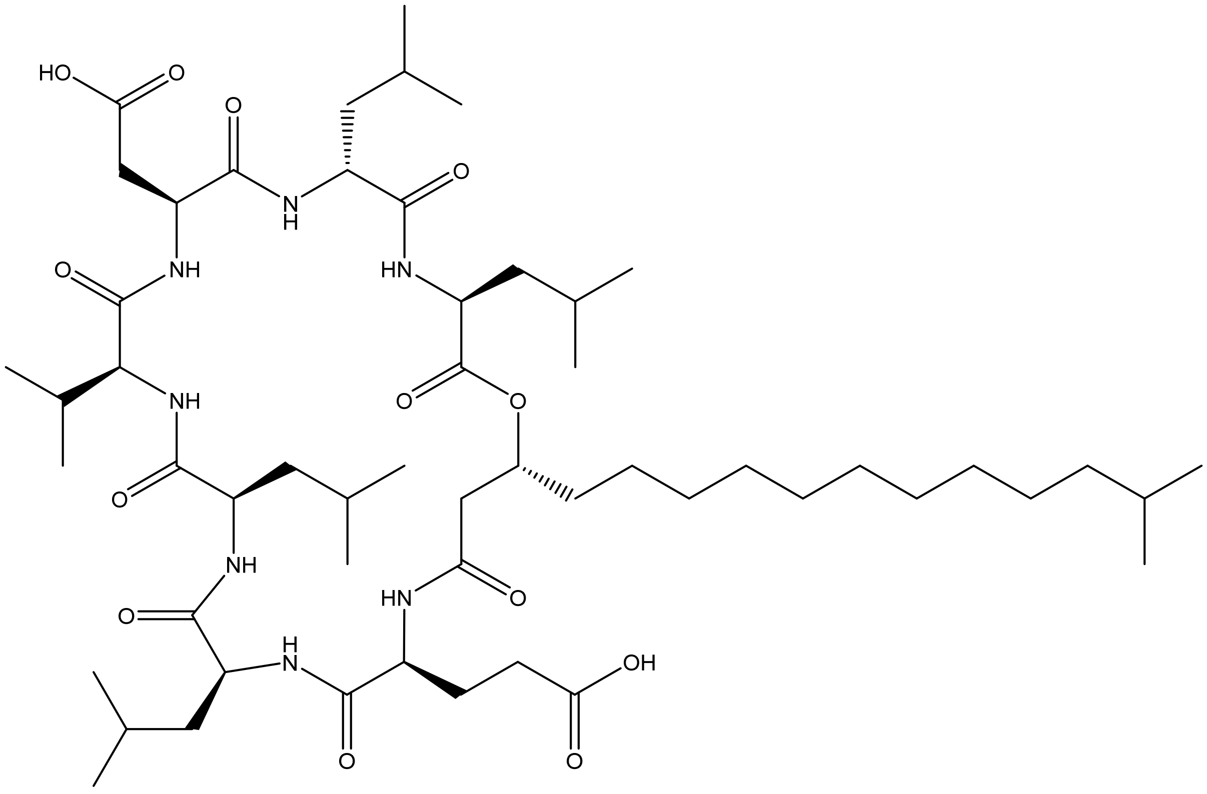

Position		δ_C	δ_H (J in Hz)
Glu1	NH	-	8.44, (d), 6.6
	CO	172.2	-

	α-C	51.1	4.04, (q), 4.8
	β-C	39.5	1.63, (m)
	γ-C	39.3	1.63, (m)
	COOH		
Leu2	NH	-	8.13 d, 7.2
	CO	173.6	-
	α-C	52.3	4.16, (m)
	β-C	24.5	1.47, (m)
	γ-C	39.8	1.50, (m)
	δ_1-C	23.7	0.87, (m)
	δ_2-C	23.4	0.86, (m)
Leu3	NH	-	7.60, (s)
	CO	157.5	-
	α-C	51.4	4.32, (q), 5.6
	β-C	24.7	1.52, (m)
	γ-C	39.0	1.26, (m)
	δ_1-C	23.8	0.86, (m)
	δ_2-C	22.9	0.82, (m)
Val4	NH	-	8.00, (s)
	CO	-	-
	α-C	51.2	4.02, (t), 7.6
	β-C	30.3	2.02, (m)
	γ_1-C	22.9	0.90, (m)
	γ_2-C	22.6	0.76, (m)
Asp5	NH	-	8.19, (s)
	CO		-
	α-C	49.9	4.55, (m)
	β-C	35.9	2.63, (t), 8.0
	COOH		
Leu6	NH	-	7.61, (s)
	CO		-
	α-C	51.6	4.33, (m)
	β-C	40.3	1.46, (m)
	γ-C	23.4	1.14, (m)
	δ_1-C	23.1	0.89, (m)
	δ_2-C	22.3	0.88, (m)
Leu7	NH	-	7.90, (s)
	CO		-
	α-C	52.3	4.14, (m)
	β-C	30.1	1.25, (m)
	γ-C	41.6	1.56, (m)
	δ_1-C	23.1	0.83, (m)
	δ_2-C	22.7	0.80, (m)
Fatty acid	C1		
part		171.4	-
	C2	41.7	2.38, (m)
	C3	71.6	5.06, (m)
	C4	33.7	1.51, (m)
	C5-15	22.3	0.80, (m)
	C16	29.7	2.03, (m)
	C17	22.1	0.80, (m)
	C18	24.3	0.81, (m)

Figure S1 (+)-LRESIMS spectrum of compound 1

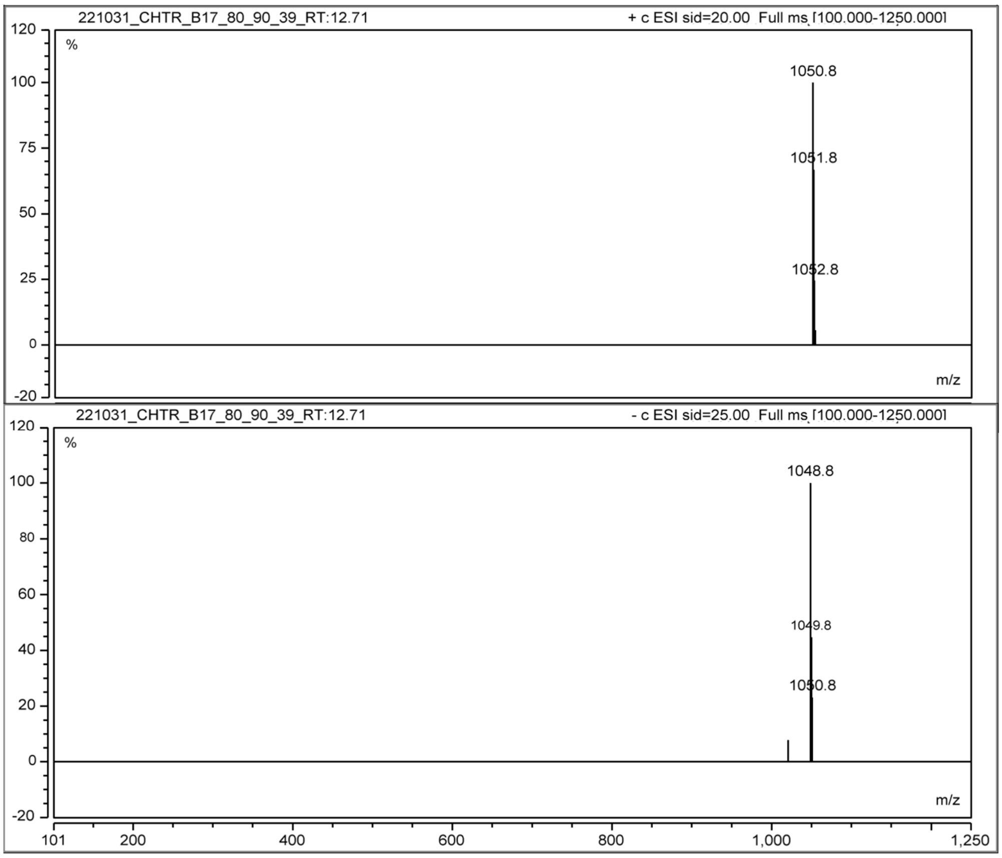

Figure S2 ^{1}H NMR (800 MHz, DMSO) spectrum of compound 1

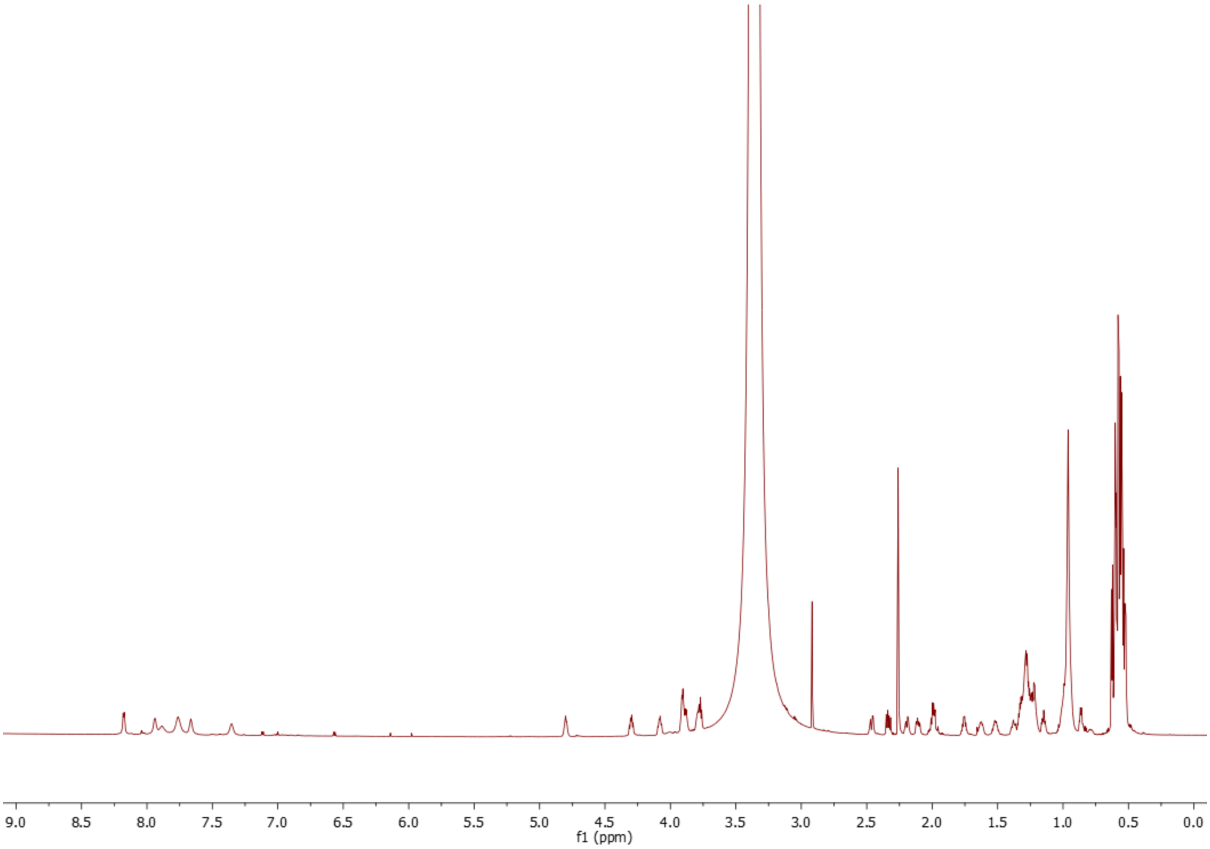

Figure S3 $^{1}H-^{1}H$ COSY spectrum of compound 1 in DMSO

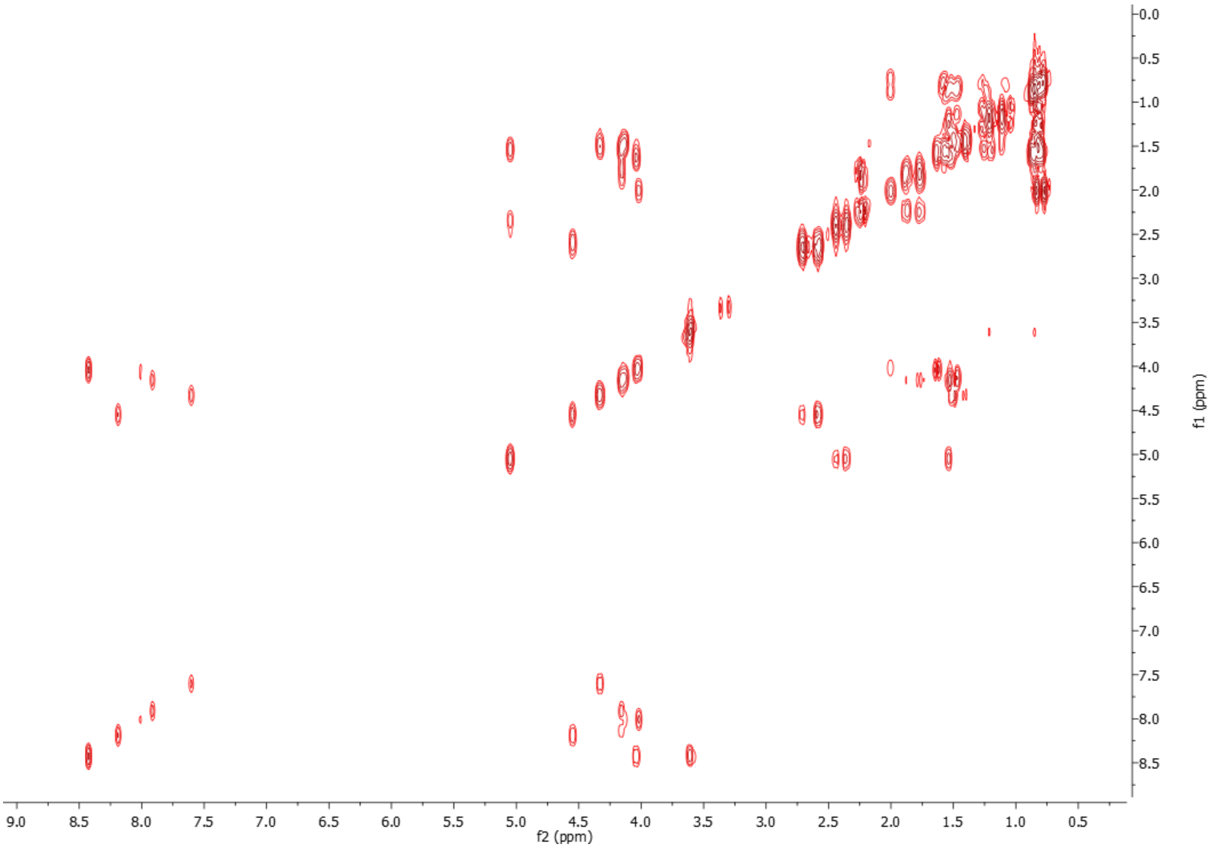

Figure S4 HSQC spectrum of compound 1 in DMSO

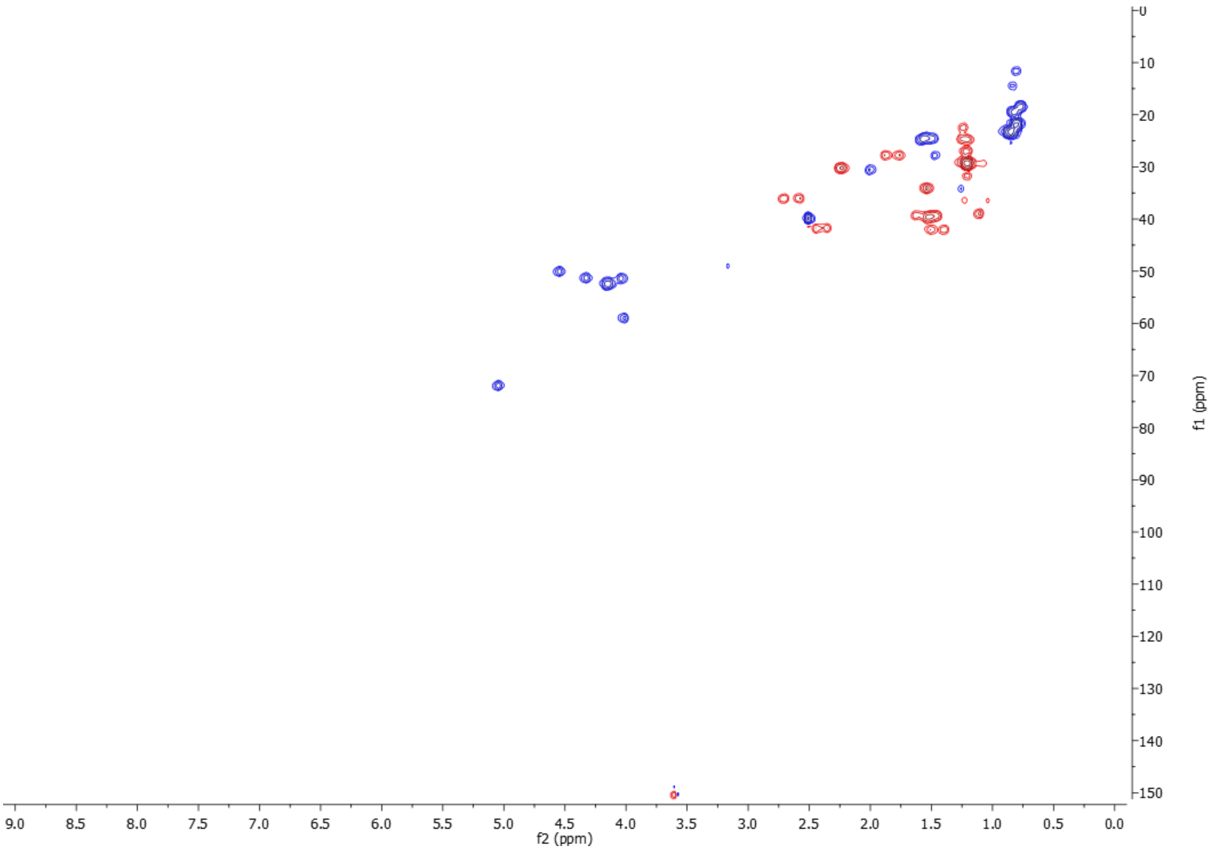

Figure S5 HMBC spectrum of compound 1 in DMSO

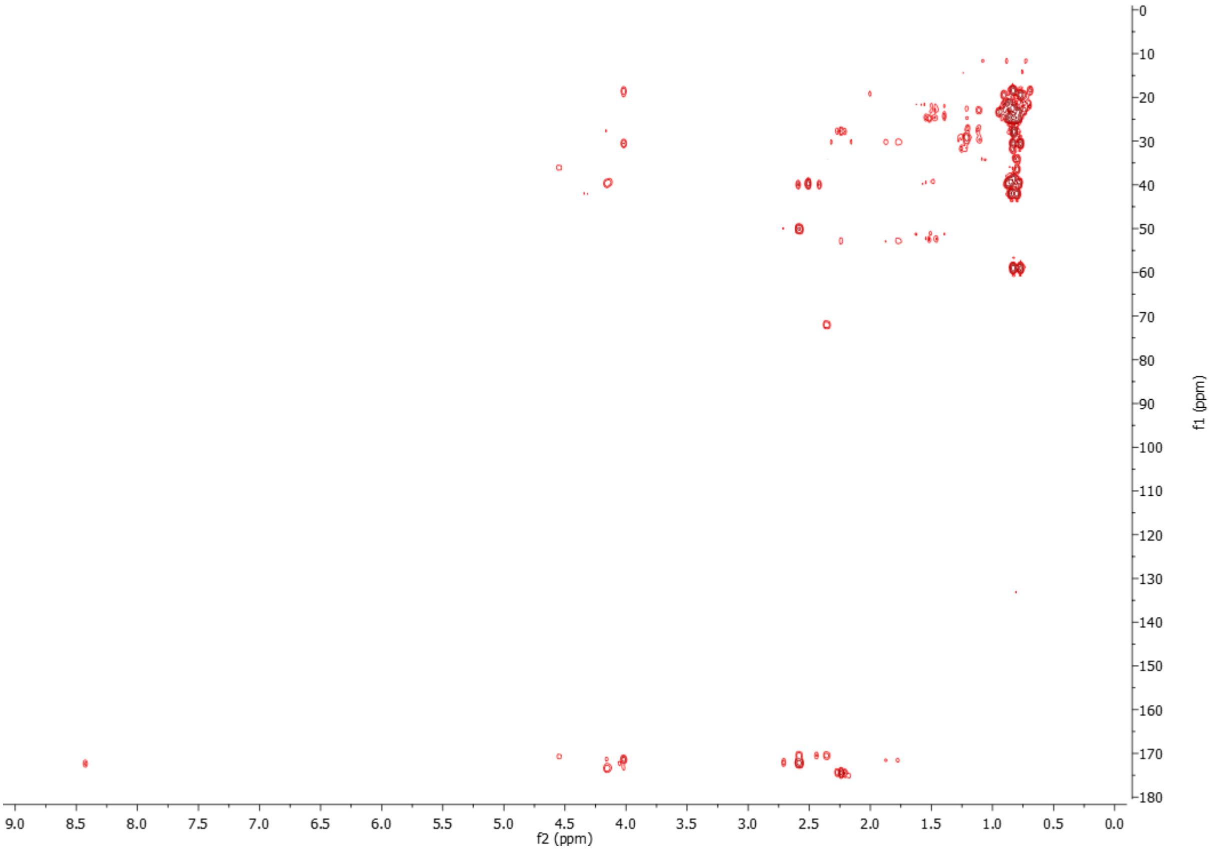

Table S2 Experimental NMR data of compound 2 in DMSO - d_6 at 25°C

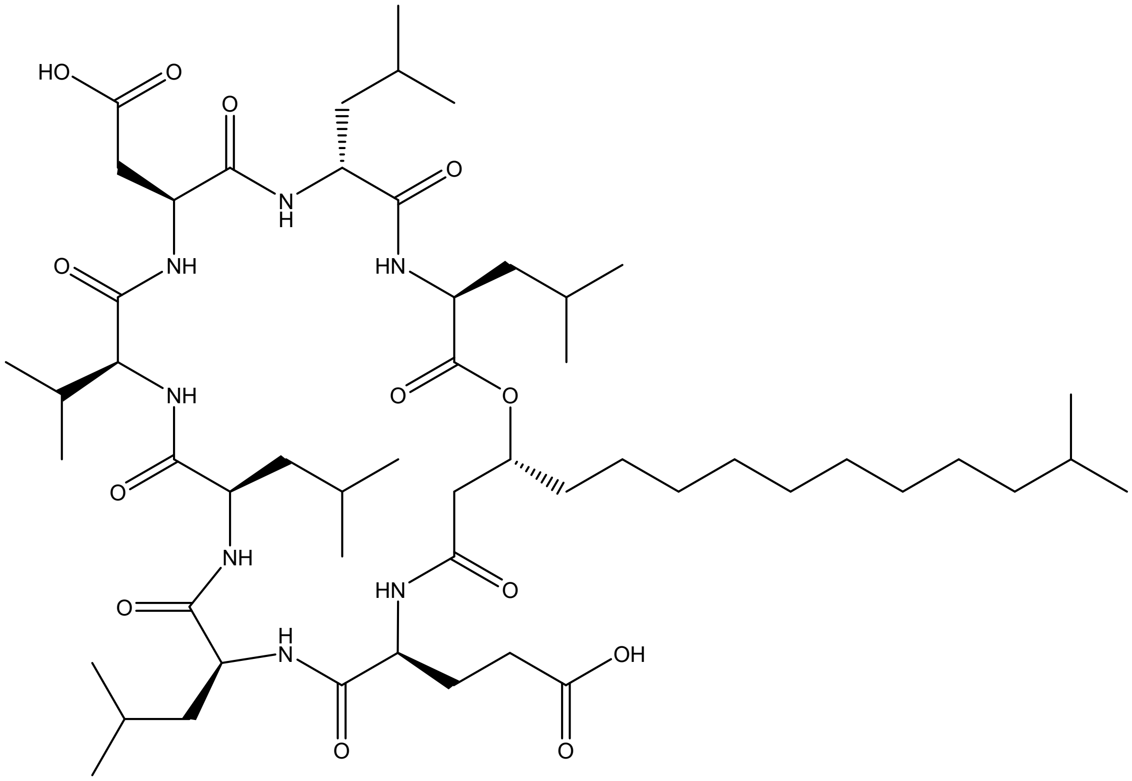

Position		δ_C	δ_H (*J* in Hz)
Glu1	NH	-	8.14, (d), 6.6
	CO	170.5	-
	α-C	50.2	4.55, (q), 4.8
	β-C	36.3	2.58, (m)
	γ-C	40.3	2.74, (dd), 4.8/12.8
	COOH	172.3	12.0
Leu2	NH	-	8.44, d, 7.2
	CO	172.4	-
	α-C	51.5	4.08, (m)
	β-C	39.0	1.66 (m)
	γ-C	28.5	1.55 (m)
	δ_1-C	22.5	0.83, (m)
	δ_2-C	22.5	0.83, (m)
Leu3	NH	-	7.60, (s)
	CO	172.2	-
	α-C	51.2	4.37, (q), 5.6
	β-C	42.1	1.50, (m)
	γ-C	39.8	1.60, (m)
	δ_1-C	22.5	0.87, (m)
	δ_2-C	22.5	0.87, (m)
Val4	NH	-	8.05, (s)
	CO	171.1	-
	α-C	59.0	4.04, (t), 7.6
	β-C	30.6	2.01, (m)
	γ_1-C	18.6	0.81, (m)
	γ_2-C	19.5	0.89, (m)

Asp5	NH	-	7.99, (s)
	CO	173.4	-
	α-C	52.7	4.17, (m)
	β-C	30.1	1.80, (m) / 2.24, (t), 8.0
	COOH	174.5	12.33, (s)
Leu6	NH	-	7.81, (s)
	CO	171.1	-
	α-C	52.8	4.17, (m)
	β-C	39.9	1.50 (m)/1.61 (m)
	γ-C	21.9	0.80
	δ_1-C	18.1	0.81 (m)
	δ_2-C	19.2	0.88 (m)
Leu7	NH	-	7.81, (s)
	CO	173.4	-
	α-C	52.8	4.17, (m)
	β-C	39.9	1.50 (m)/1.61 (m)
	γ-C	21.9	0.80
	δ_1-C	18.1	0.81 (m)
	δ_2-C	19.2	0.88 (m)
Fatty acid part	C1	170.3	-
	C2	41.9	2.50 (m)/2.33 (m)
	C3	71.8	5.08, (m)
	C4	39.3	1.53, (m)
	C5-15	26.1	0.84, (m)
	C16	24.6	1.62, (m)
	C17	23.2	0.91, (m)
	C18	23.2	0.91, (m)

Figure S6 (+)-LRESIMS spectrum of compound 2

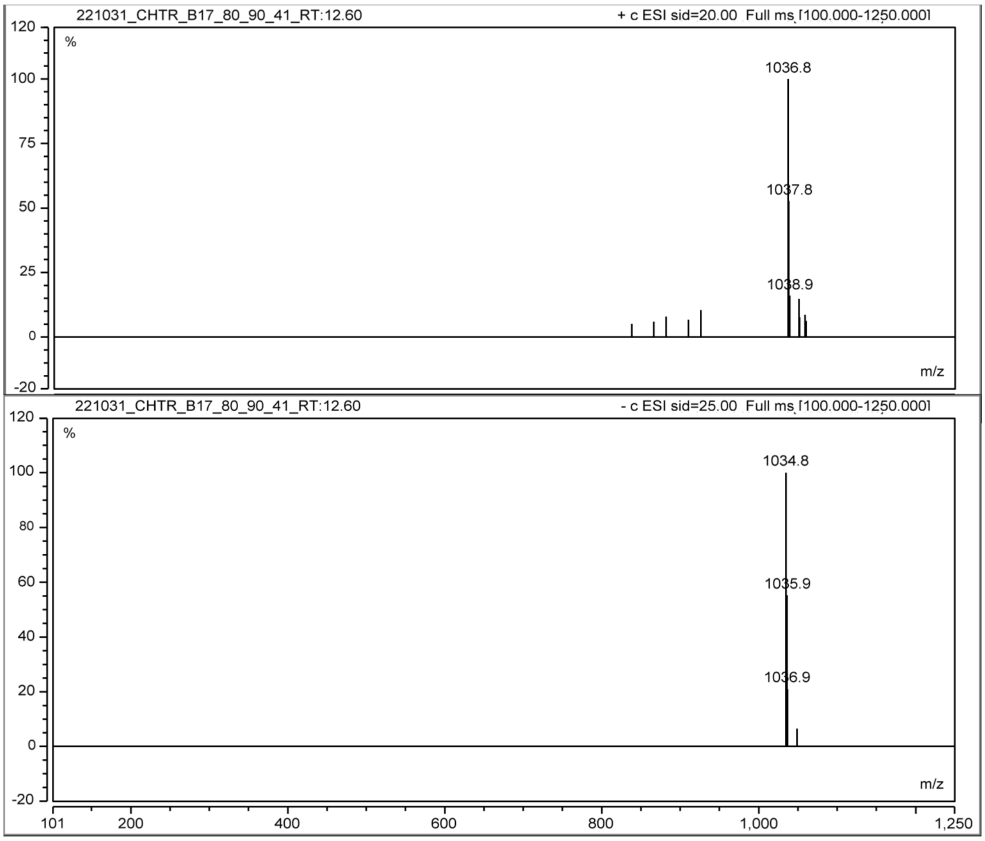

Figure S7 ^{1}H NMR (800 MHz, DMSO) spectrum of compound 2

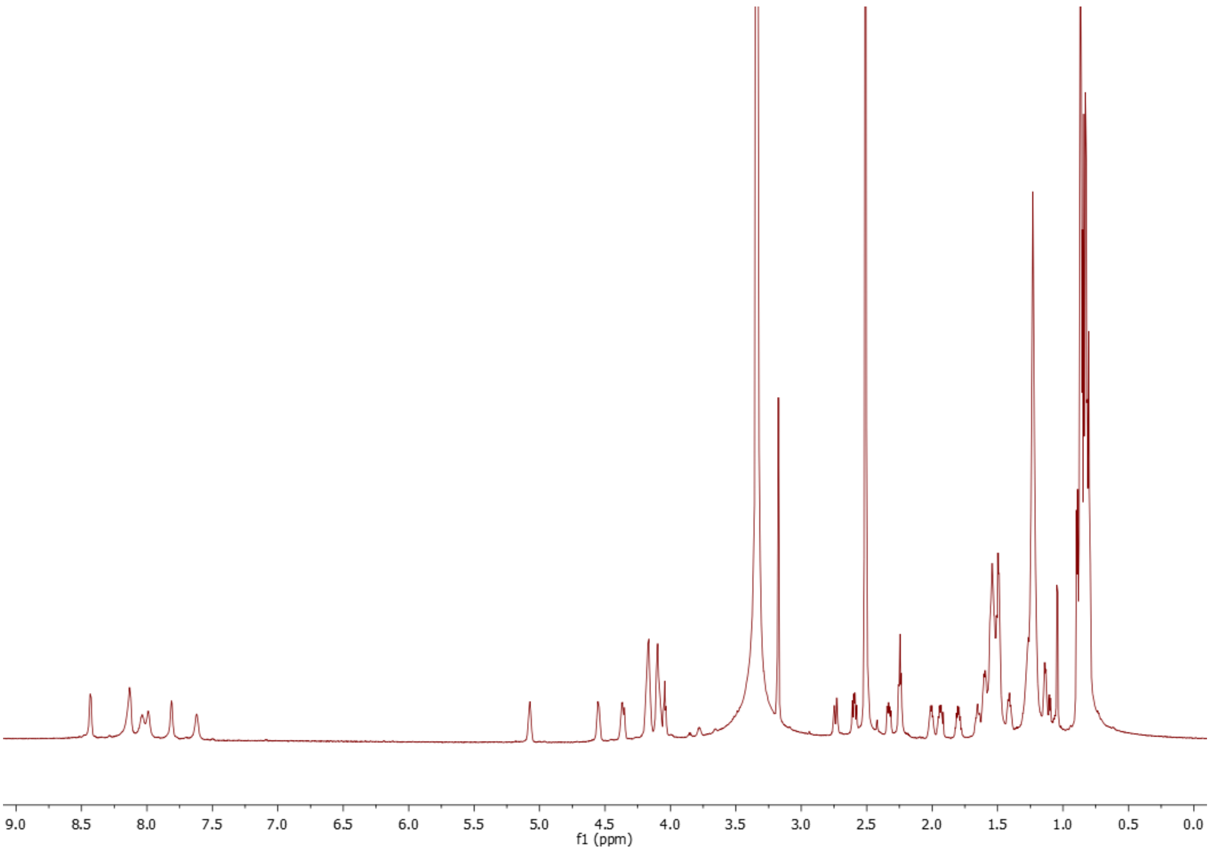

Figure S8 ^{1}H－^{1}H COSY spectrum of compound 2 in DMSO

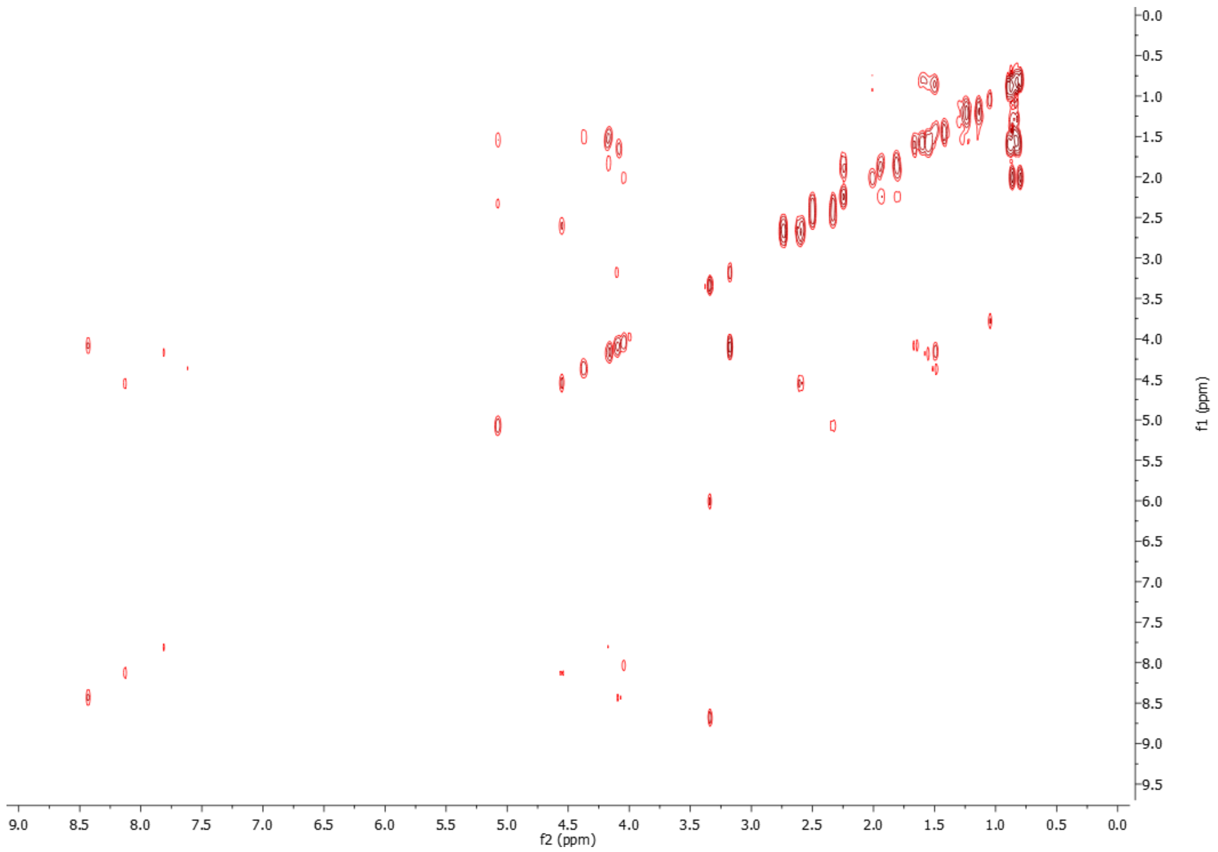

Figure S9 HSQC spectrum of compound 2 in DMSO

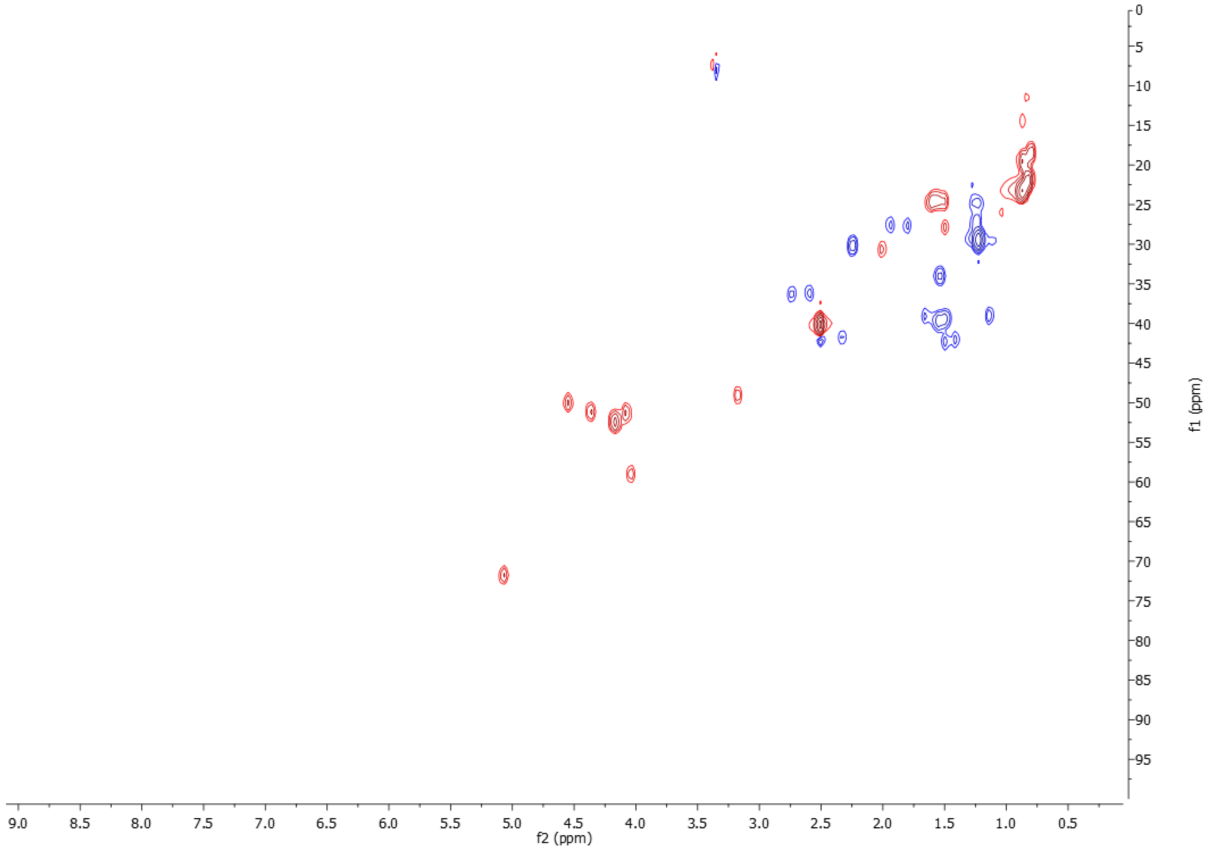

Figure S10 HMBC spectrum of compound 2 in DMSO

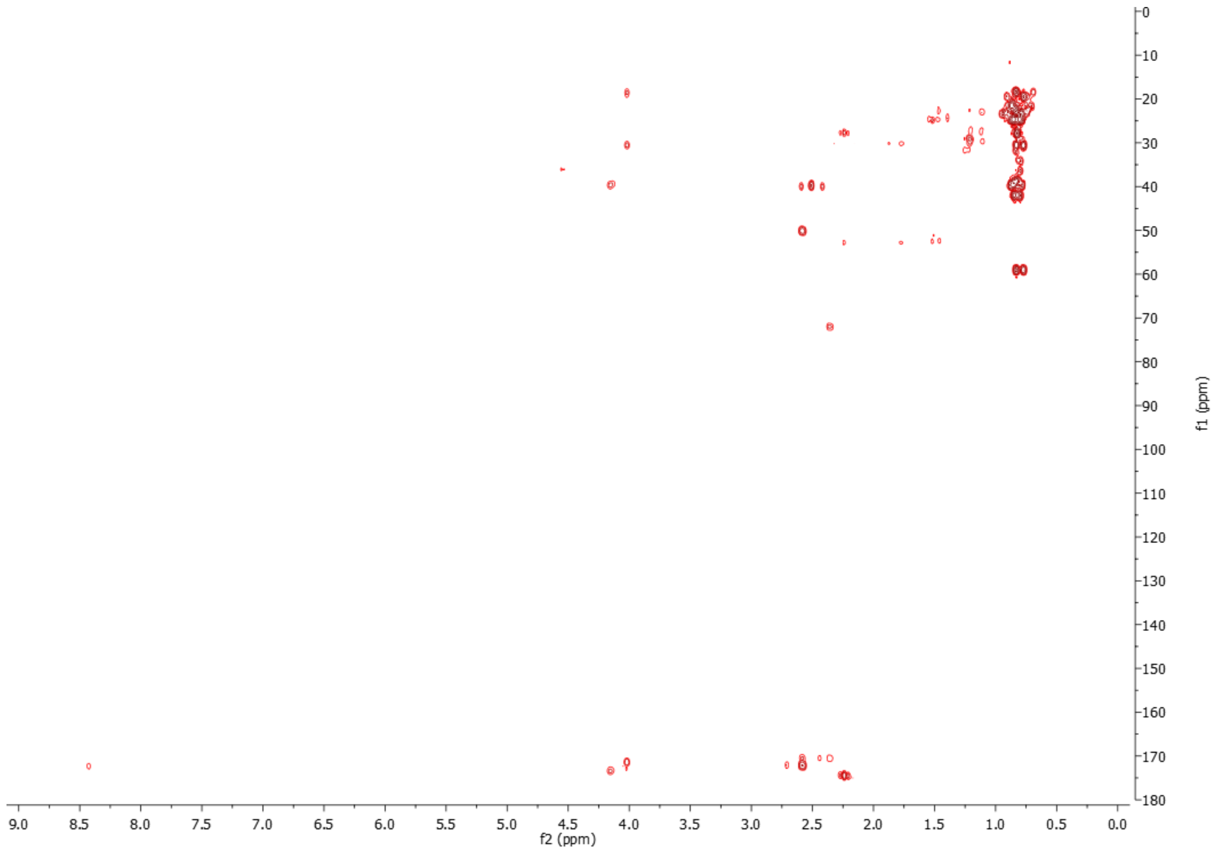

Table S3 Experimental NMR data of compound 3 in DMSO - *d*6 at 25°C

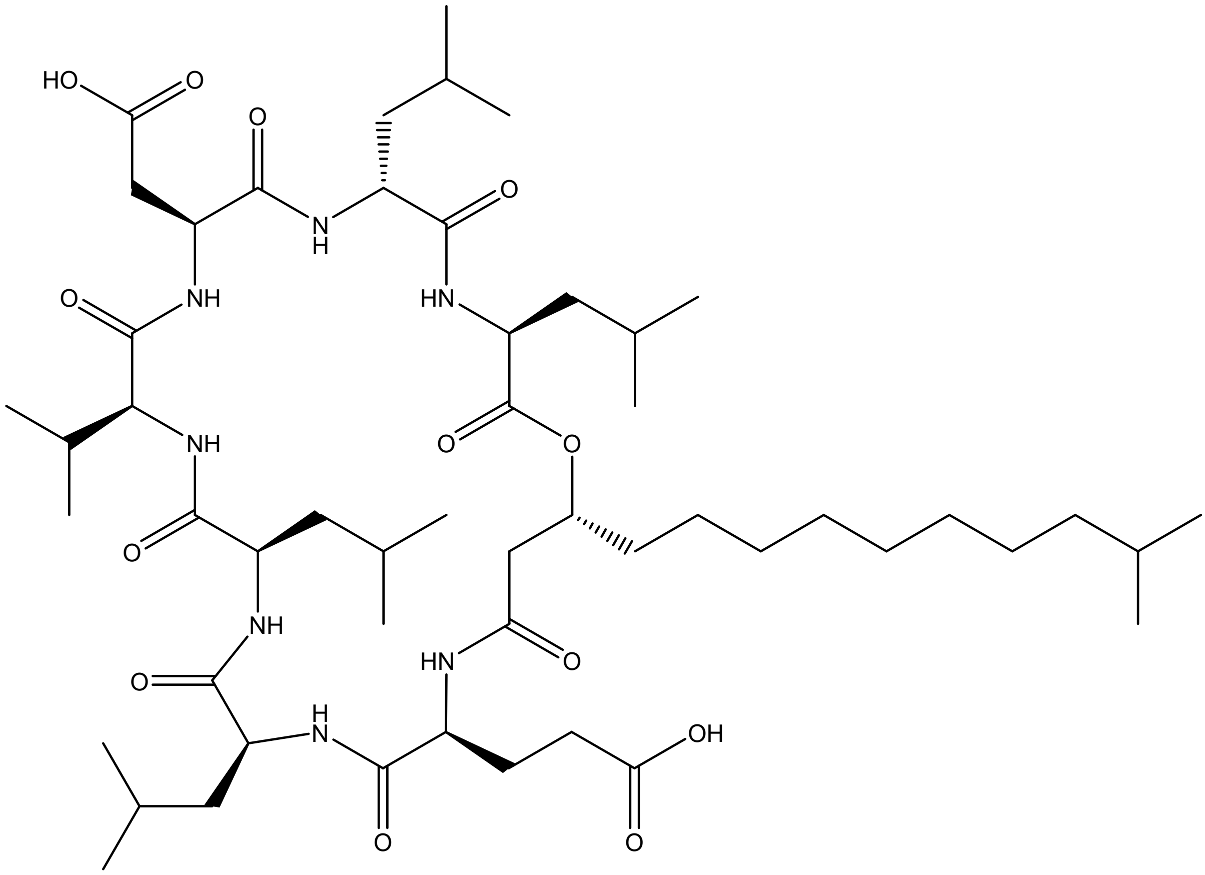

Position		δ_C	δ_H (*J* in Hz)
Glu1	NH	-	8.42, (d), 6.6
	CO	172.2	-
	α-C	51.1	4.04, (q), 4.8
	β-C	39.5	1.63, (m)
	γ-C	39.3	1.63, (dd), 4.8/12.8
	COOH	172.3	12.0
Leu2	NH	-	8.14, d, 7.2
	CO	173.6	-
	α-C	52.3	4.16, (m)
	β-C	24.5	1.47, (m)
	γ-C	39.8	1.50, (m)
	δ_1-C	23.7	0.87, (m)
	δ_2-C	23.4	0.86, (m)
Leu3	NH	-	7.60, (s)
	CO	157.5	-
	α-C	51.4	4.35, (q), 5.6
	β-C	24.7	1.52, (m)
	γ-C	39.0	1.26, (m)
	δ_1-C	23.8	0.86, (m)
	δ_2-C	22.9	0.82, (m)
Val4	NH	-	8.00, (s)
	CO	-	-
	α-C	51.2	4.04, (t), 7.6
	β-C	30.3	2.02, (m)

	γ_1-C	22.9	0.90, (m)
	γ_2-C	22.6	0.76, (m)
Asp5	NH	-	8.16, (s)
	CO		-
	α-C	49.9	4.54, (m)
	β-C	35.9	2.60, (m)
	COOH		12.33 (s)
Leu6	NH	-	7.60, (s)
	CO		-
	α-C	51.6	4.36, (m)
	β-C	40.3	1.46, (m)
	γ-C	23.4	1.14, (m)
	δ_1-C	23.1	0.89, (m)
	δ_2-C	22.3	0.88, (m)
Leu7	NH	-	7.88, (s)
	CO		-
	α-C	52.3	4.16, (m)
	β-C	30.1	1.25, (m)
	γ-C	41.6	1.56, (m)
	δ_1-C	23.1	0.83, (m)
	δ_2-C	22.7	0.80, (m)
Fatty acid part	C1	171.4	-
	C2	41.7	2.34, (m)
	C3	71.6	5.06, (m)
	C4	33.7	1.51, (m)
	C5-15	22.3	0.80, (m)
	C16	29.7	2.03, (m)
	C17	22.1	0.80, (m)
	C18	24.3	0.81, (m)

Figure S11 (+)-LRESIMS spectrum of compound 3

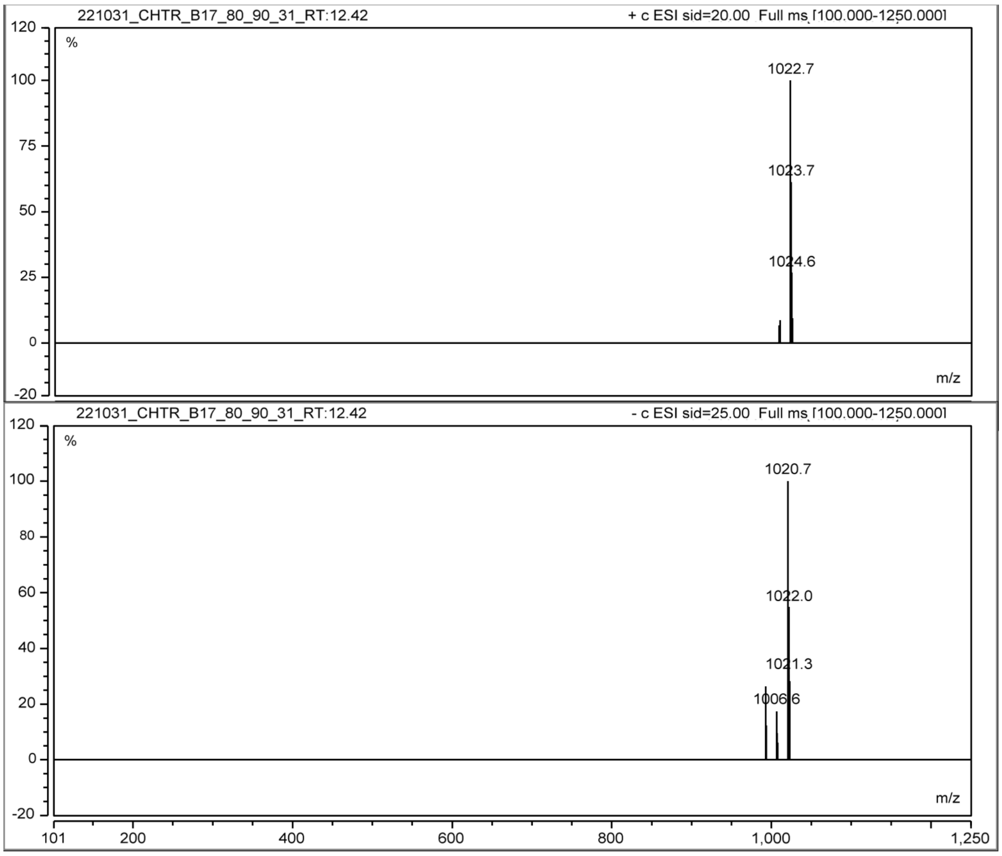

Figure S12 ^{1}H NMR (800 MHz, DMSO) spectrum of compound 3

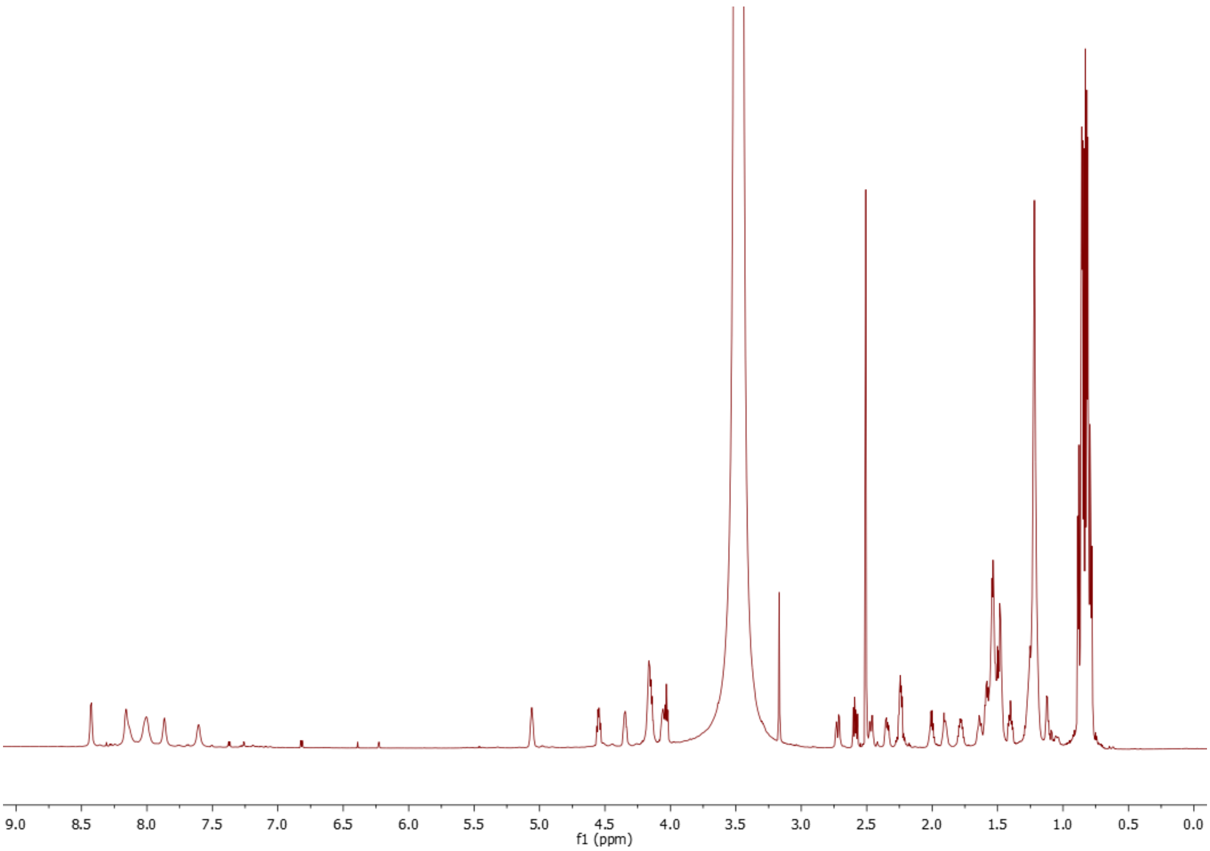

Figure S13 $^{1}H-^{1}H$ COSY spectrum of compound 3 in DMSO

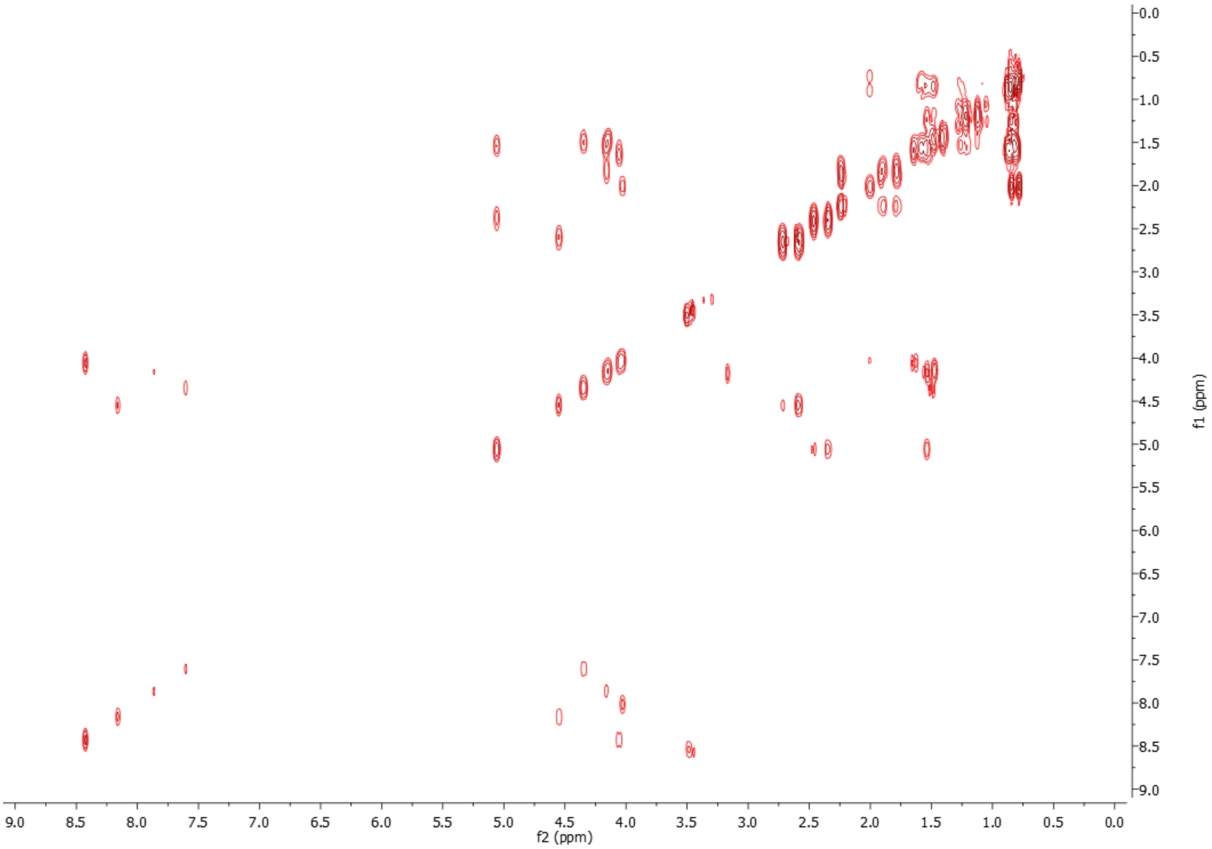

Figure S14 HSQC spectrum of compound 3 in DMSO

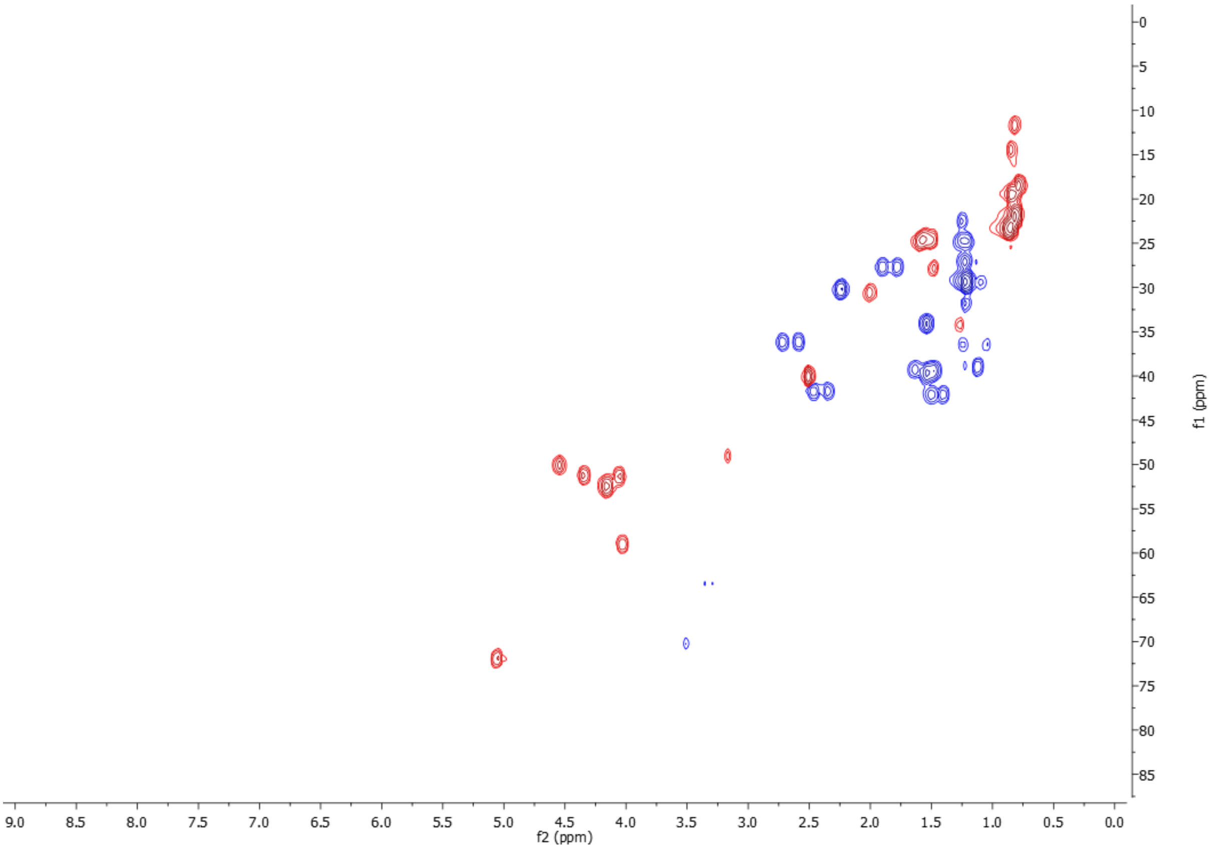

Figure S15 HMBC spectrum of compound 3 in DMSO

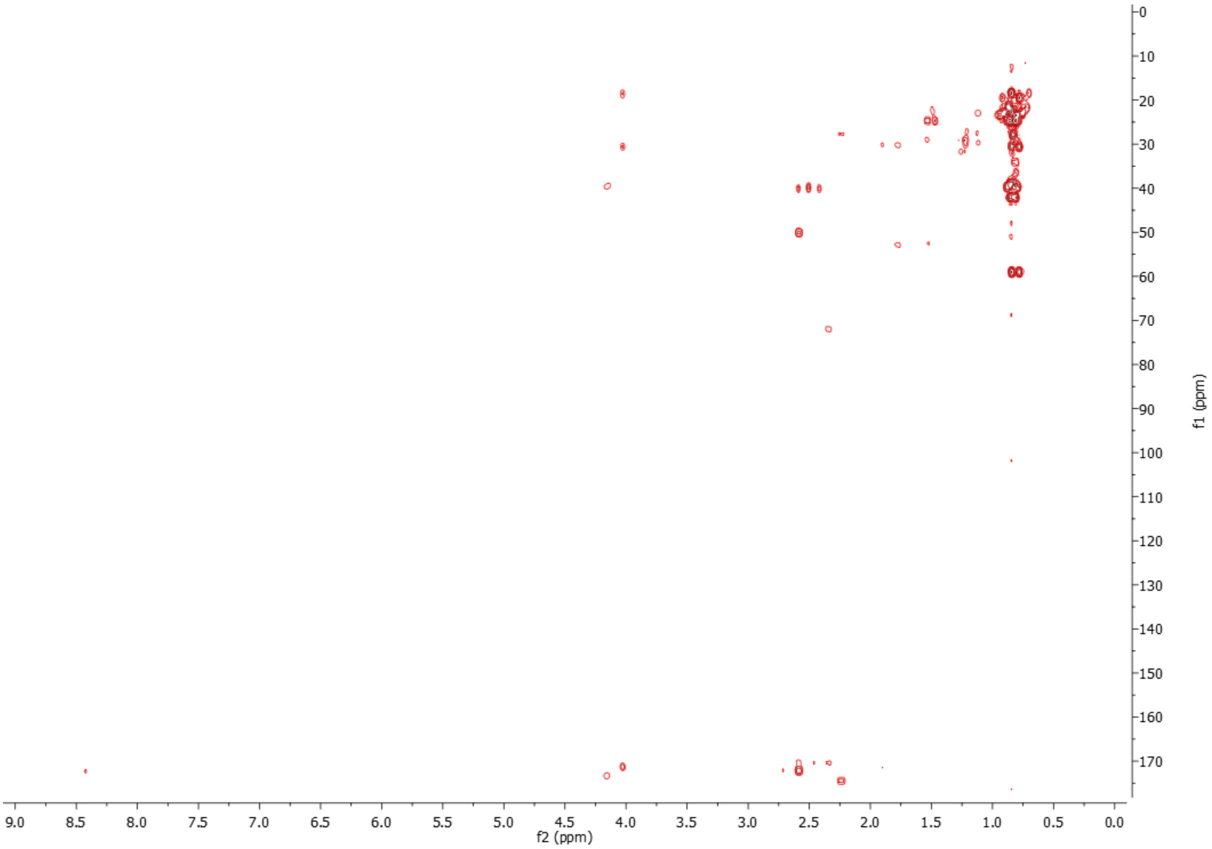

Table S4 Experimental NMR data of compound 4 in DMSO - *d*6 at 25°C

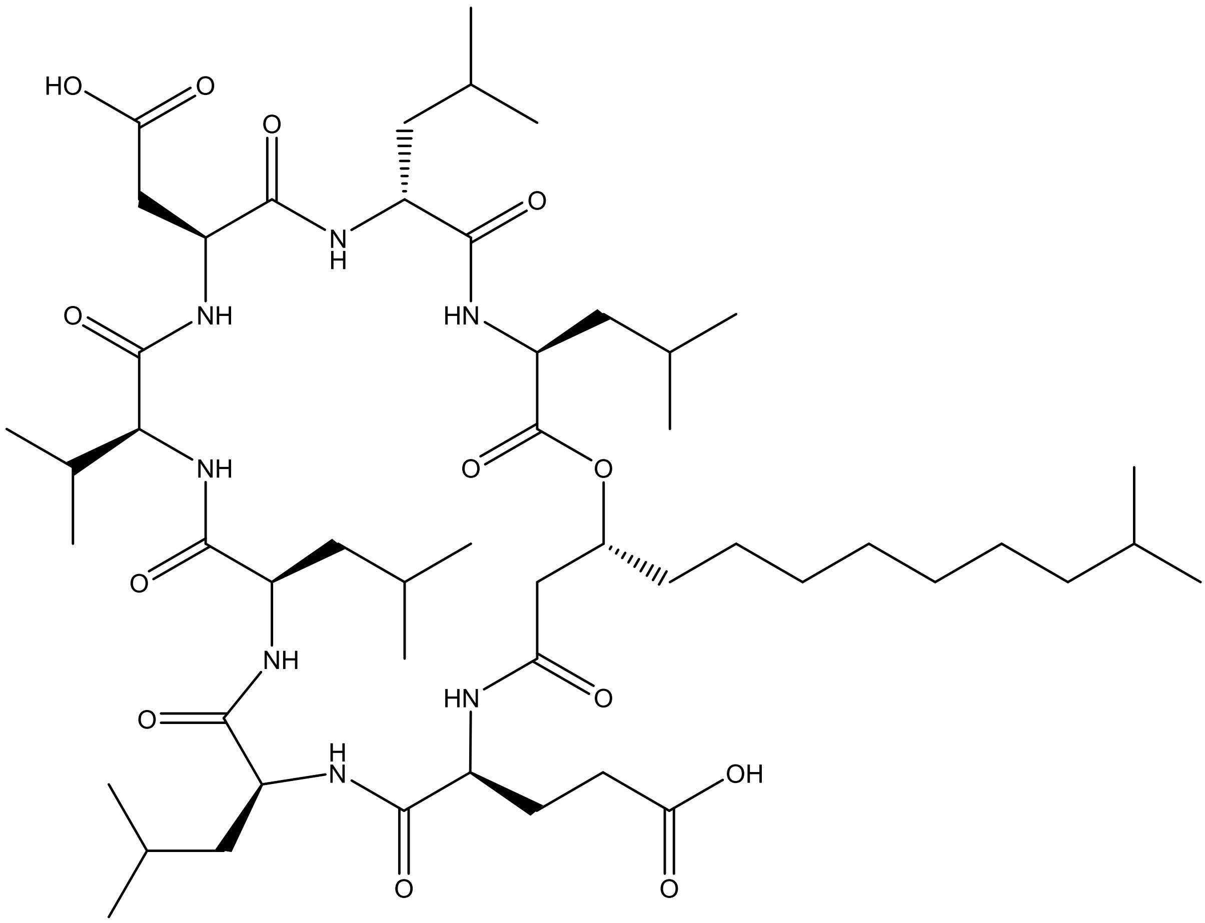

Position		δ_C	δ_H (J in Hz)
Glu1	NH	-	8.44, (d), 6.6
	CO	172.2	-
	α-C	51.1	4.04, (q), 4.8
	β-C	39.5	1.63, (m)
	γ-C	39.3	1.63, (dd), 4.8
	COOH		12.0
Leu2	NH	-	8.13, (d), 7.2
	CO	173.6	-
	α-C	52.3	4.16, (m)
	β-C	24.5	1.47, (m)
	γ-C	39.8	1.50, (m)
	δ_1-C	23.7	0.87, (m)
	δ_2-C	23.4	0.86, (m)
Leu3	NH	-	7.61, (s)
	CO	157.5	-
	α-C	51.4	4.35, (q), 5.6
	β-C	24.7	1.53, (m)
	γ-C	39.0	1.21, (m)
	δ_1-C	23.8	0.86, (m)
	δ_2-C	22.9	0.82, (m)

Val4	NH	-	8.00, (s)
	CO	-	-
	α-C	51.2	4.04, (t), 7.6
	β-C	30.3	2.00, (m)
	γ_1-C	22.9	0.90, (m)
	γ_2-C	22.6	0.76, (m)
Asp5	NH	-	8.17, (s)
	CO		-
	α-C	49.9	4.55, (m)
	β-C	35.9	2.60, (m)
	COOH		12.33, (s)
Leu6	NH	-	7.61, (s)
	CO		-
	α-C	51.6	4.34, (m)
	β-C	40.3	1.47, (m)
	γ-C	23.4	1.13, (m)
	δ_1-C	23.1	0.89, (m)
	δ_2-C	22.3	0.88, (m)
Leu7	NH	-	7.88, (s)
	CO		-
	α-C	52.3	4.16, (m)
	β-C	30.1	1.22, (m)
	γ-C	41.6	1.56, (m)
	δ_1-C	23.1	0.83, (m)
	δ_2-C	22.7	0.80, (m)
Fatty acid part	C1	171.4	-
	C2	41.7	2.35, (m)
	C3	71.6	5.06, (m)
	C4	33.7	1.53, (m)
	C5-15	22.3	0.80, (m)
	C16	29.7	2.03, (m)
	C17	22.1	0.80, (m)
	C18	24.3	0.81, (m)

Figure S16 (+)-LRESIMS spectrum of compound 4

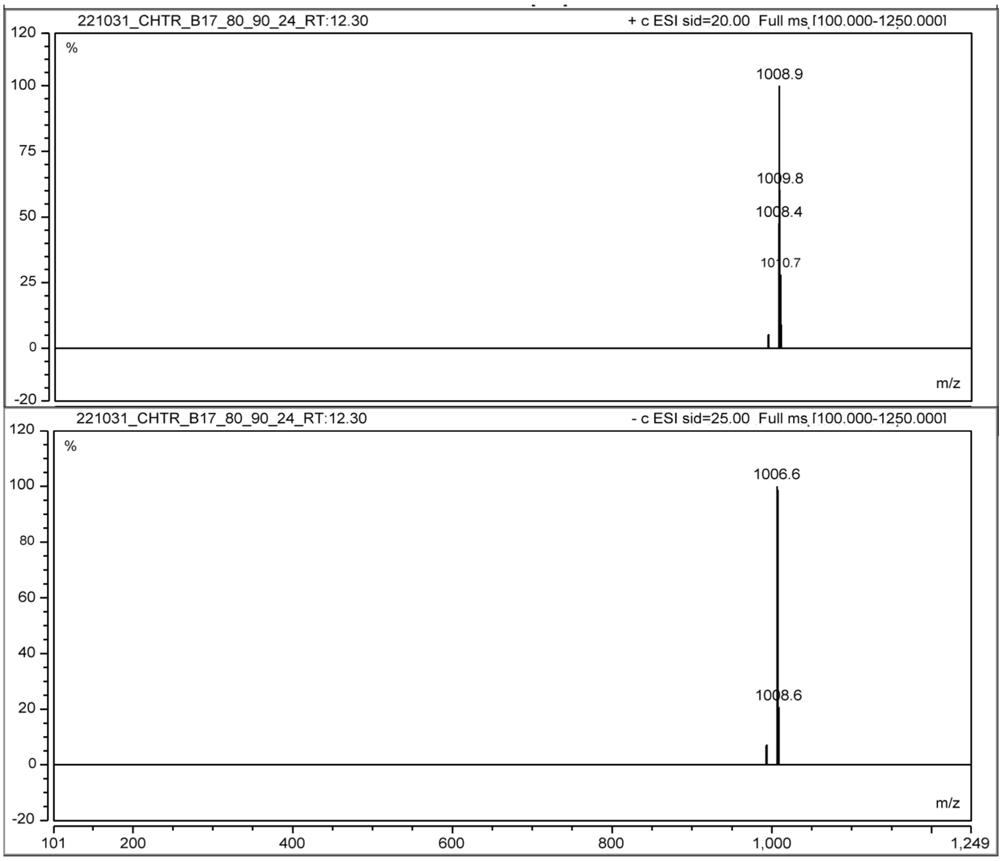

Figure S17 ^{1}H NMR (800 MHz, DMSO) spectrum of compound 4

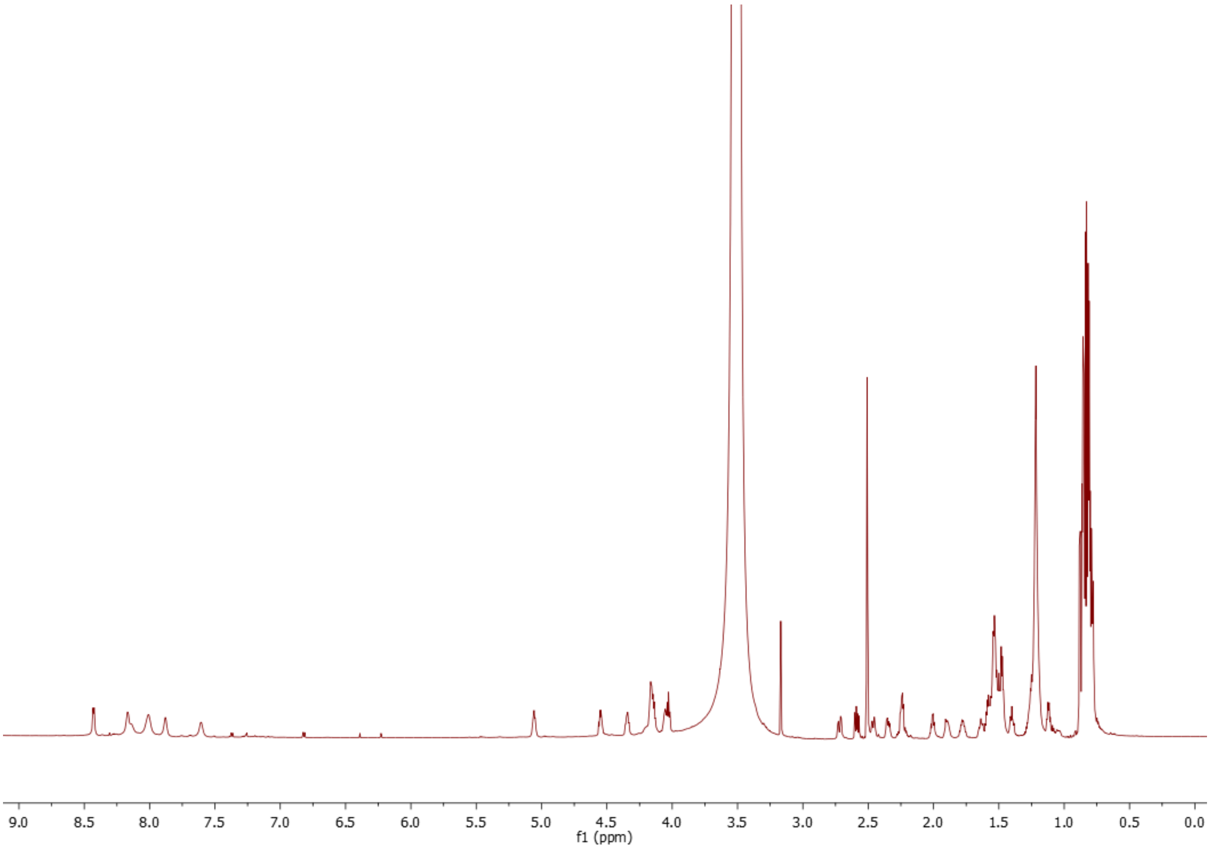

Figure S18 ^{1}H –^{1}H COSY spectrum of compound 4 in DMSO

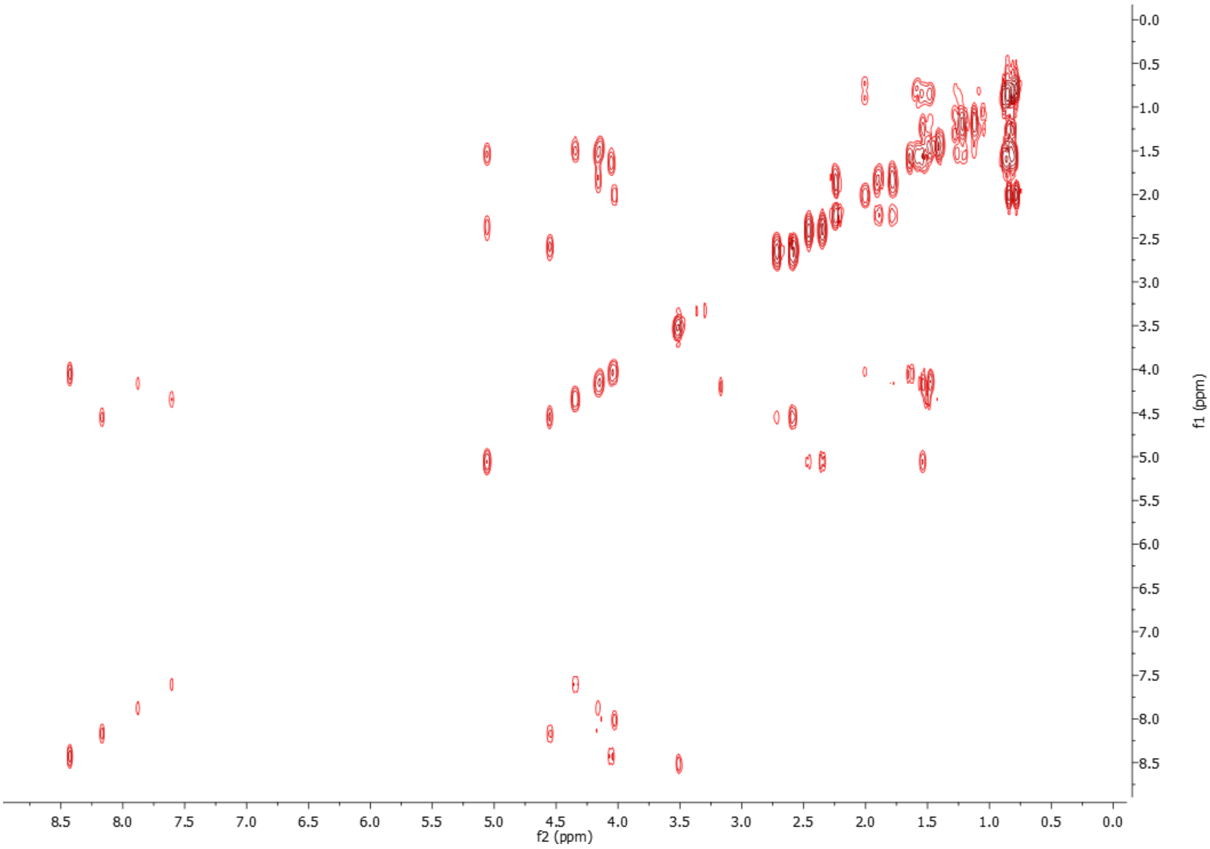

Figure S19 HSQC spectrum of compound 4 in DMSO

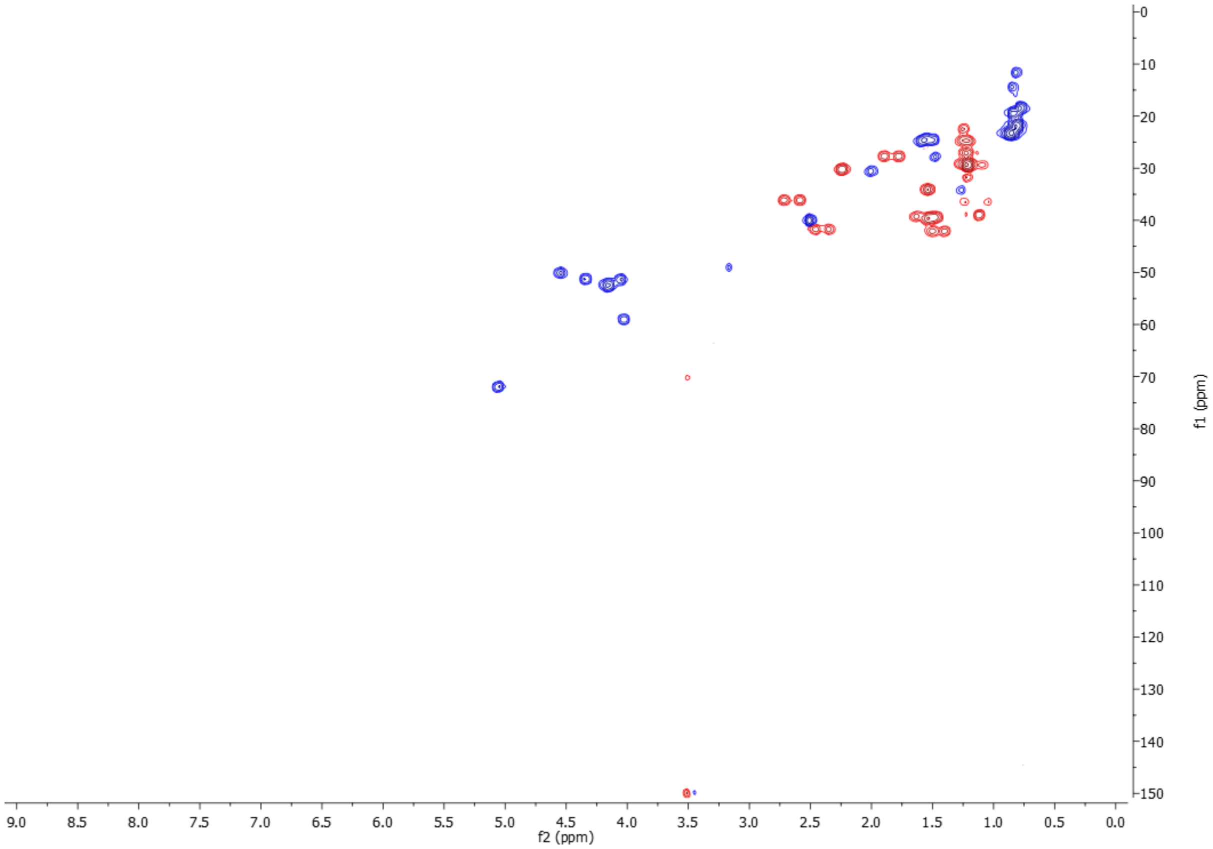

Figure S20 HMBC spectrum of compound 4 in DMSO

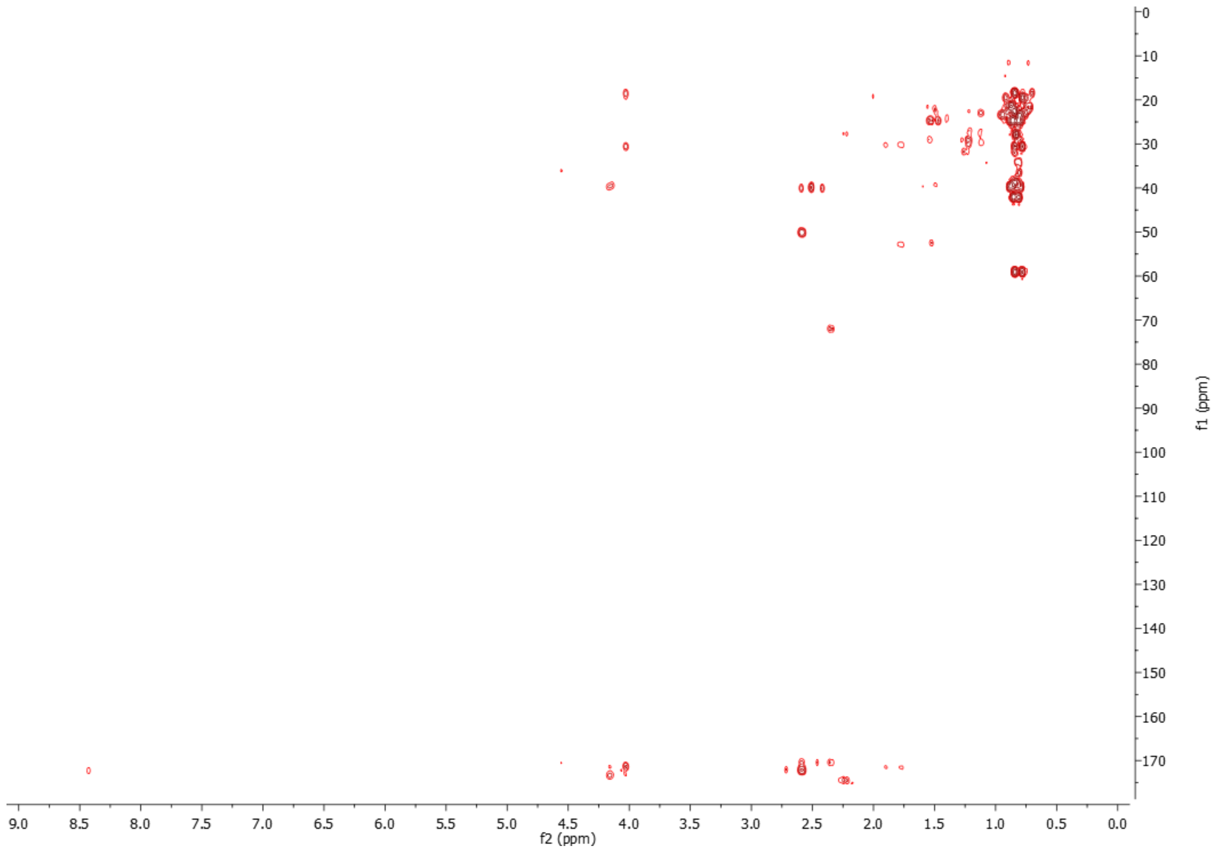

Table S5 Experimental NMR data of compound 5 in DMSO - *d*6 at 25°C

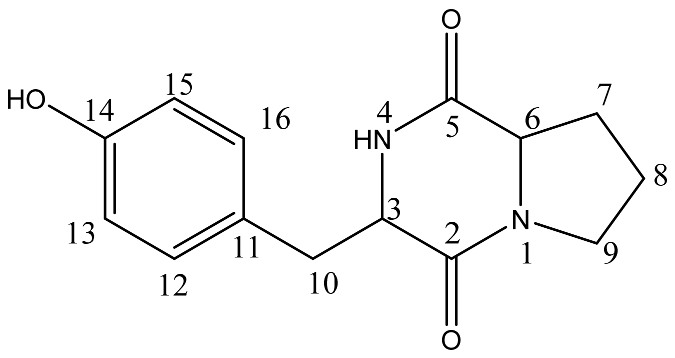

Position	δ_C	C-type	δ_H (*J* in Hz)
1	- (N)	-	-
2	165.3	CO	-
3	56.4	CH	4.25
4	-	NH	7.86
5	169.8	CO	-
6	59.0	C	4.05
7	45.0	CH2	3.43, 3.28
8	22.0	CH2	1.74
9	28.3	CH2	2.01, 1.41
10	35.5 (C-1)	CH2	2.93
11	127.1	C	-
12	131.9	CH	7.06
13	114.6	CH	6.64

14	155.6	COH	9.11
15	114.6	CH	6.64
16	131.9	CH	7.06

Figure S21 (+)-LRESIMS spectrum of compound 5

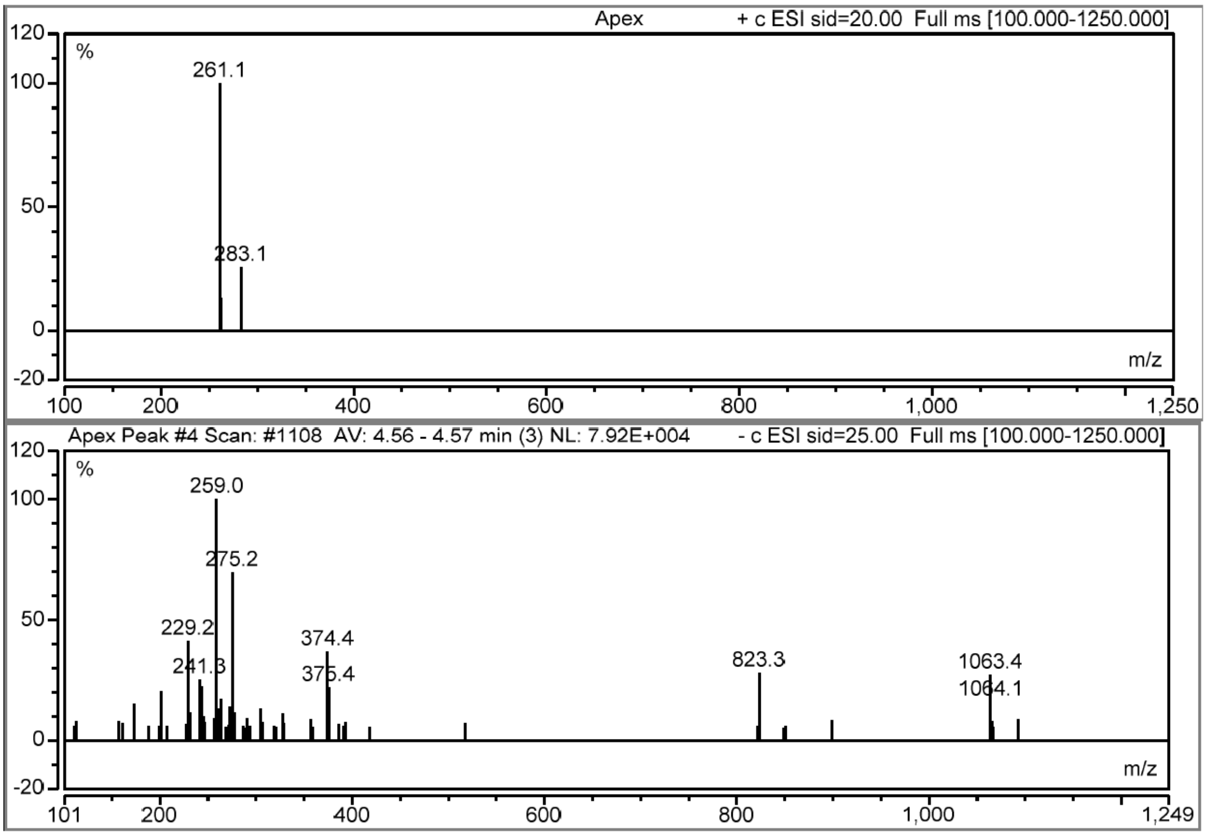

Figure S22 ^{1}H NMR (800 MHz, DMSO) spectrum of compound 5

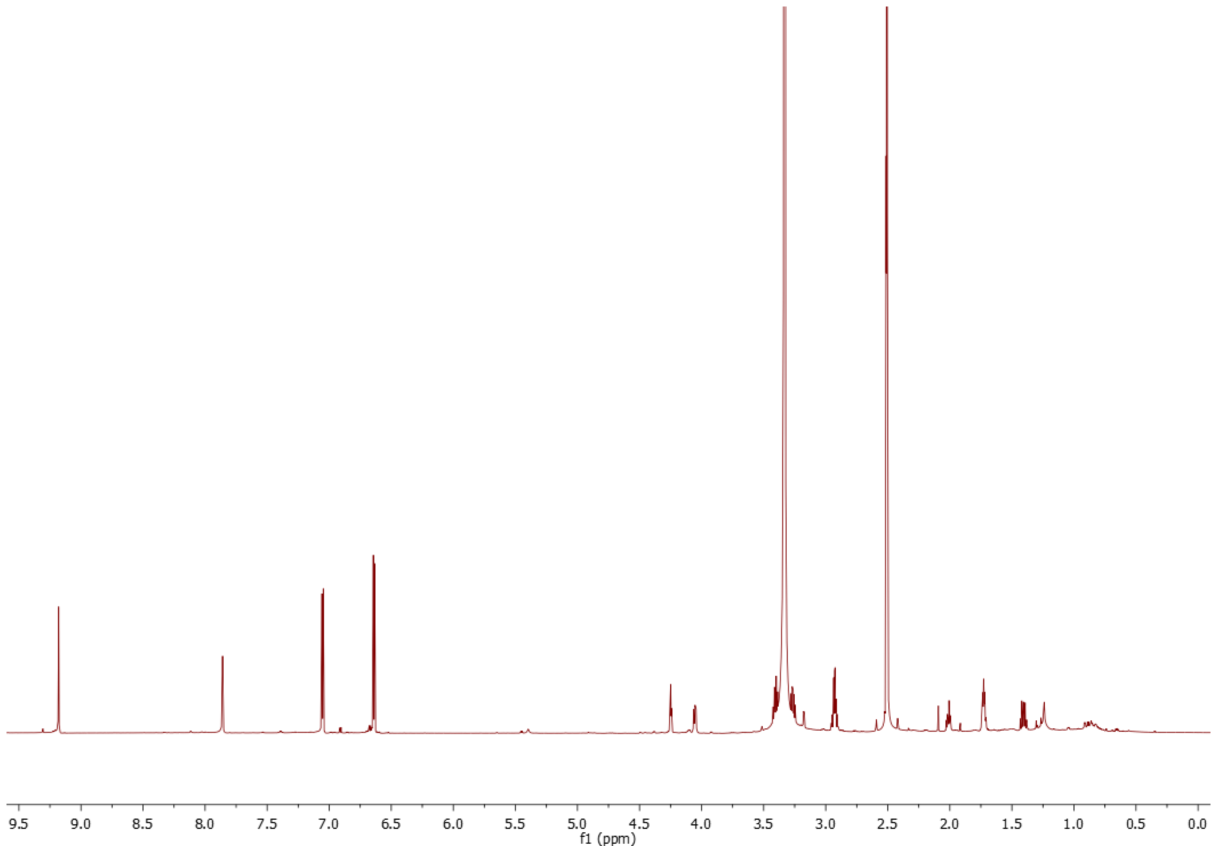

Figure S23 ^1H - ^1H COSY spectrum of compound 5 in DMSO

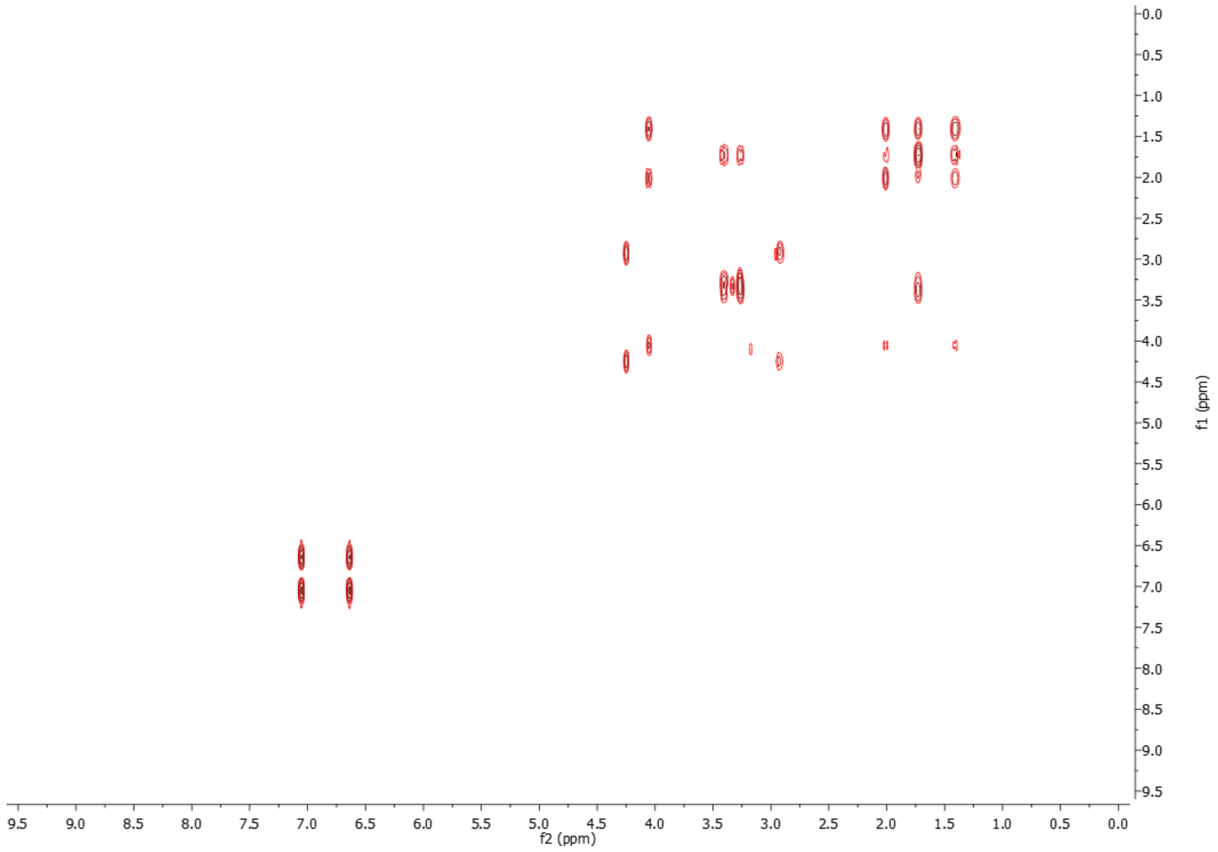

Figure S24 HSQC spectrum of compound 5 in DMSO

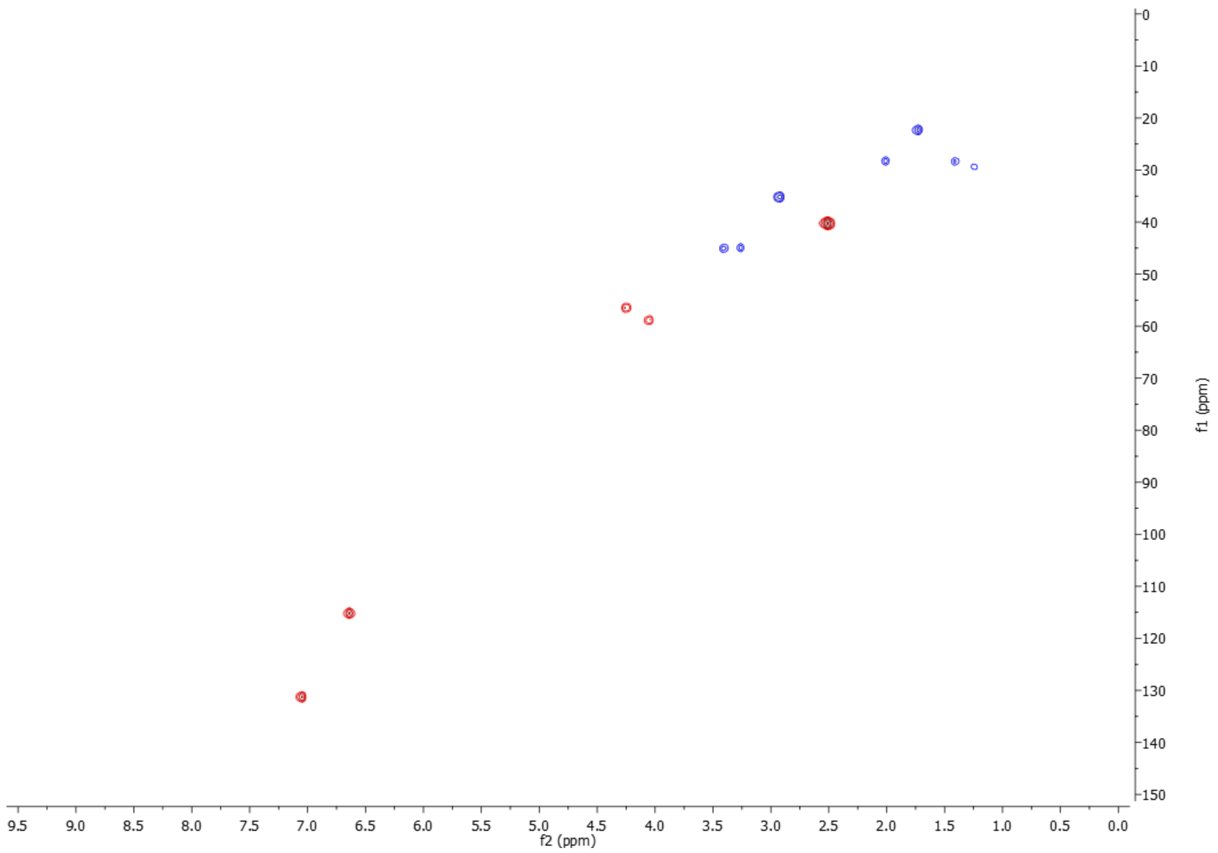

Figure S25 HMBC spectrum of compound 5 in DMSO

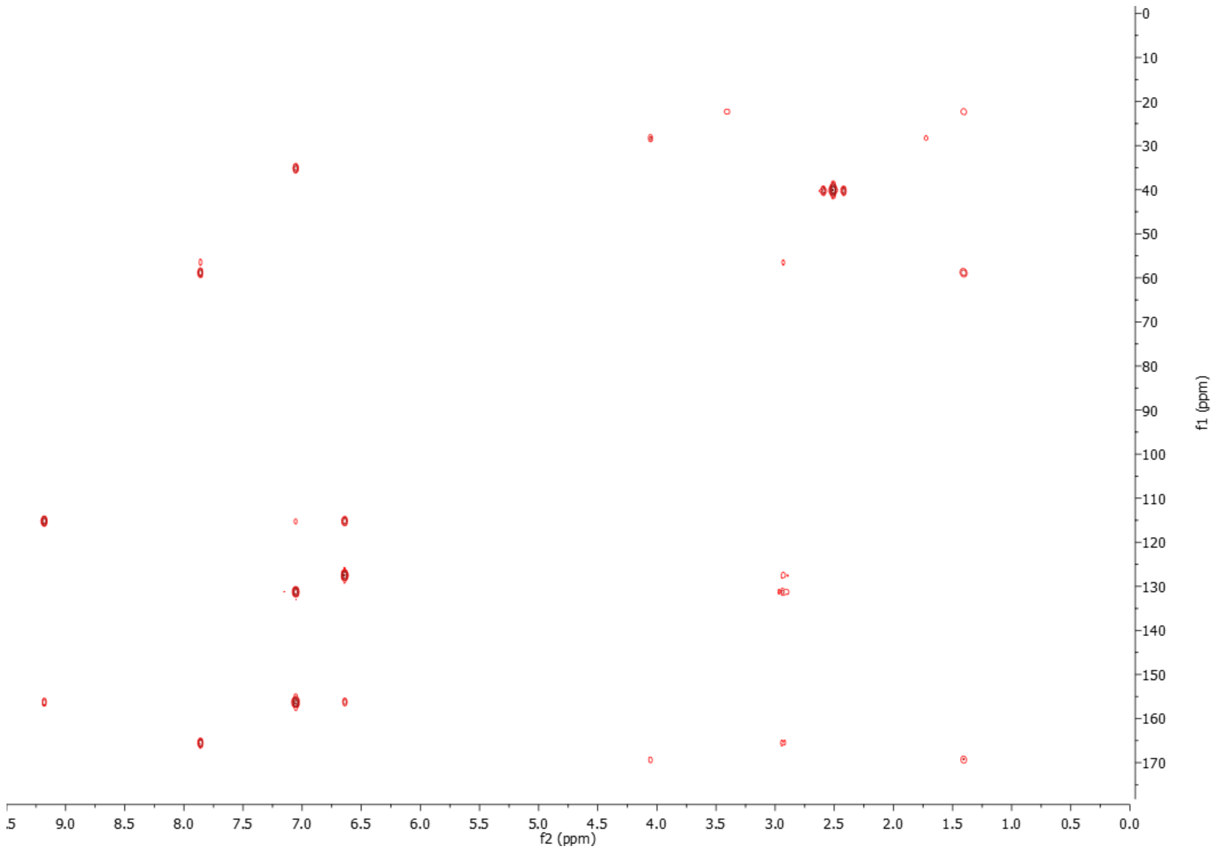

Table S6 Experimental NMR data of compound 6 in DMSO - *d*6 at 25°C

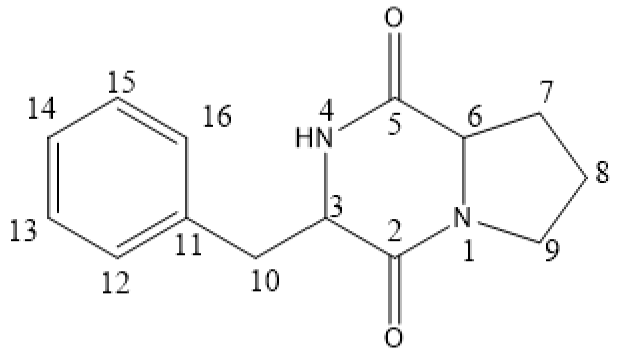

Position	δ_C	C-type	δ_H (J in Hz)
1	- (N)	-	-
2	169.3 (C-2)	C=O	-
3	(C-3)	CH	4.36
4	- (N)	-	8.00
5	165.6 (C)	-	-
6	58.7 (C)	CH	4.05
7	28.9	CH2	2.01, 1.45
8	22.6	CH2	1.72
9	44.3	CH2	3.37, 3.25
10	36.7	CH2	3.06
1	138.0	C	-
12	130.0	CH	7.28, s
13	128.0	CH	7.20, s
14	128.0	CH	7.20, s
15	128.0	CH	7.20, s
16	130.0	CH	7.20, s

Figure S26 (+)-LRESIMS spectrum of compound 6

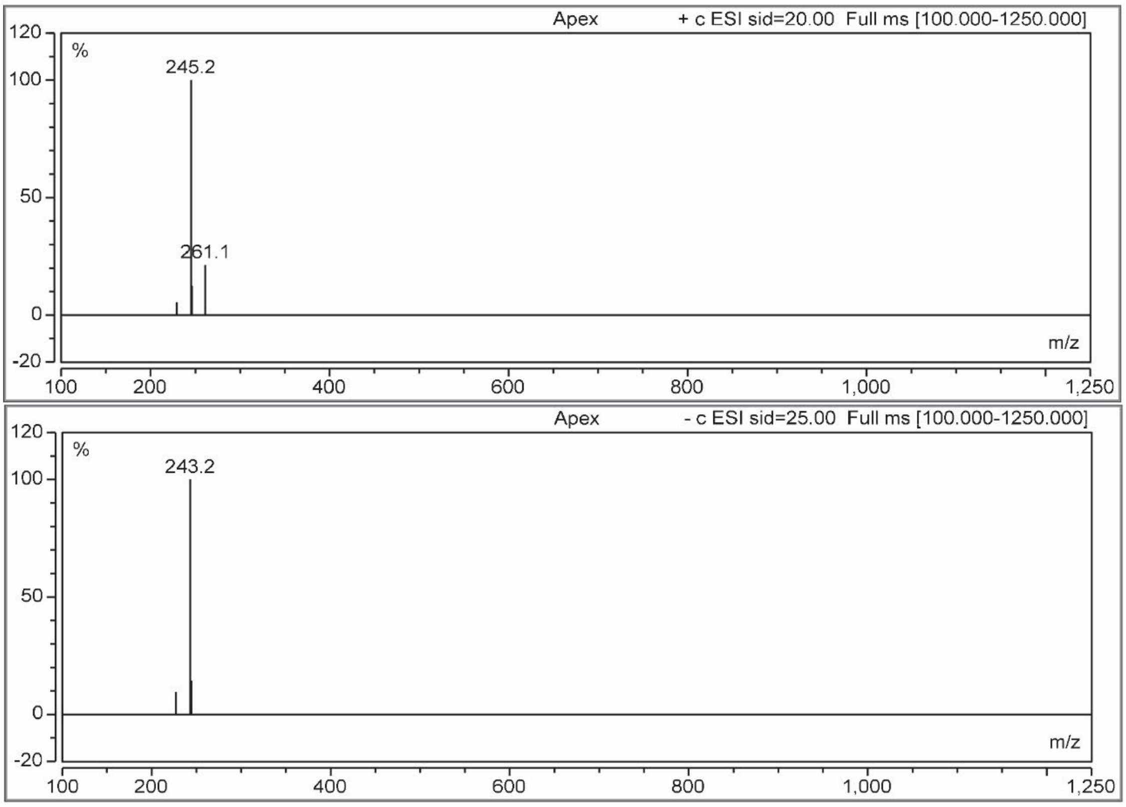

Figure S27 ^{1}H NMR (800 MHz, DMSO) spectrum of compound 6

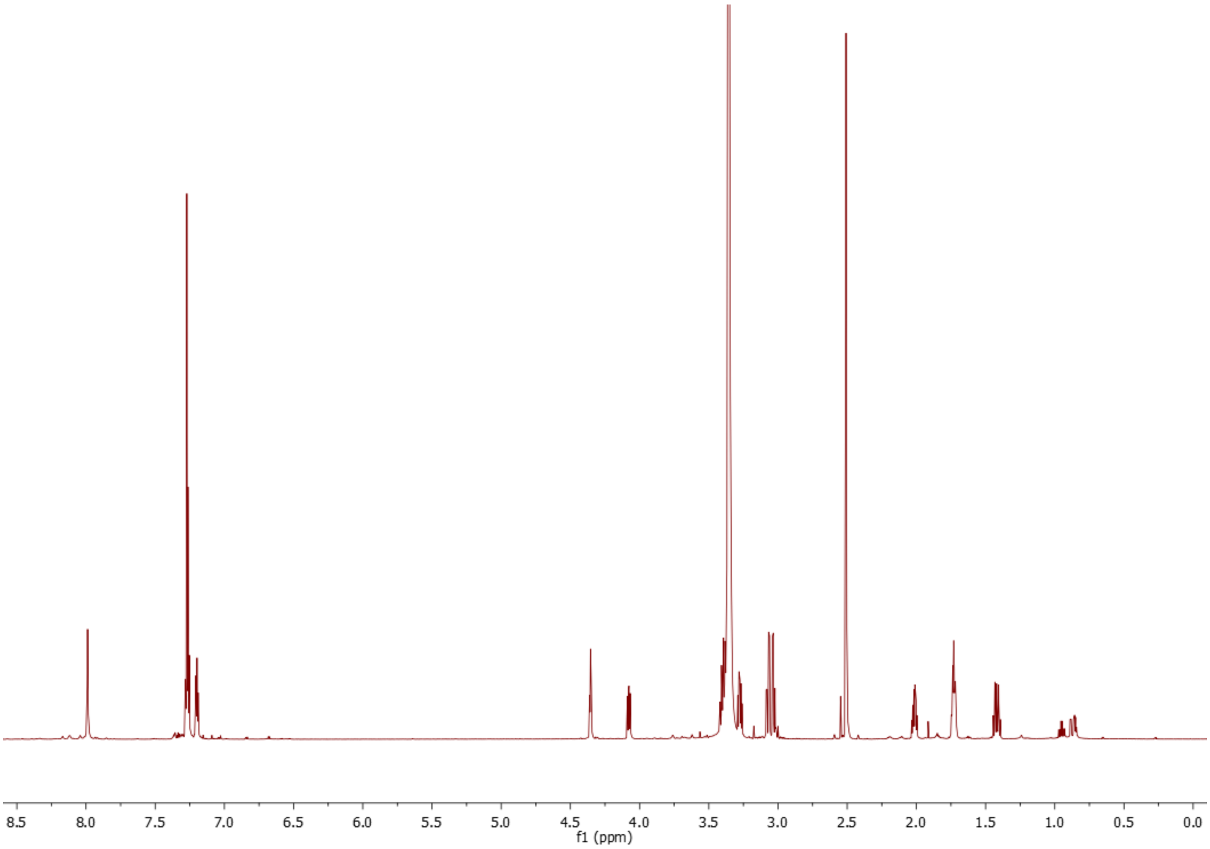

Figure S28 ^{1}H – ^{1}H COSY spectrum of compound 6 in DMSO

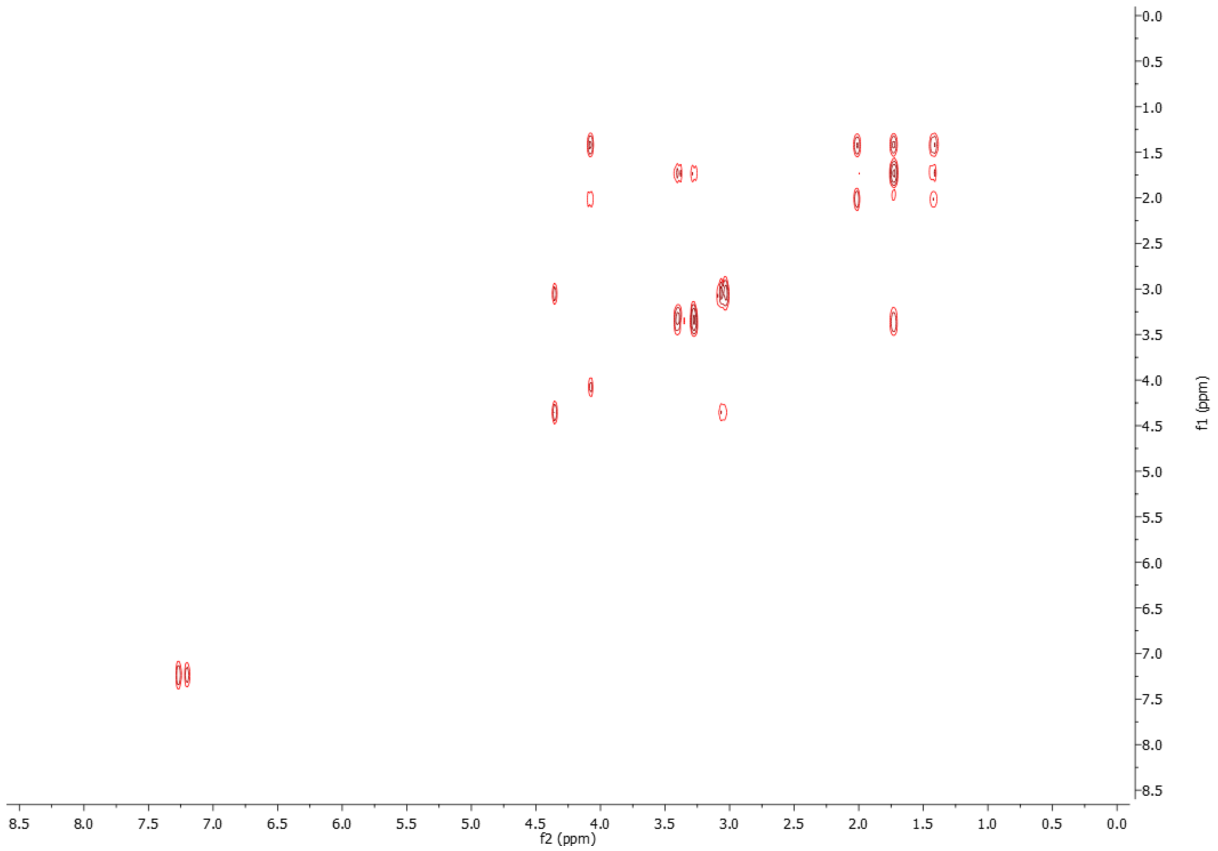

Figure S29 HSQC spectrum of compound 6 in DMSO

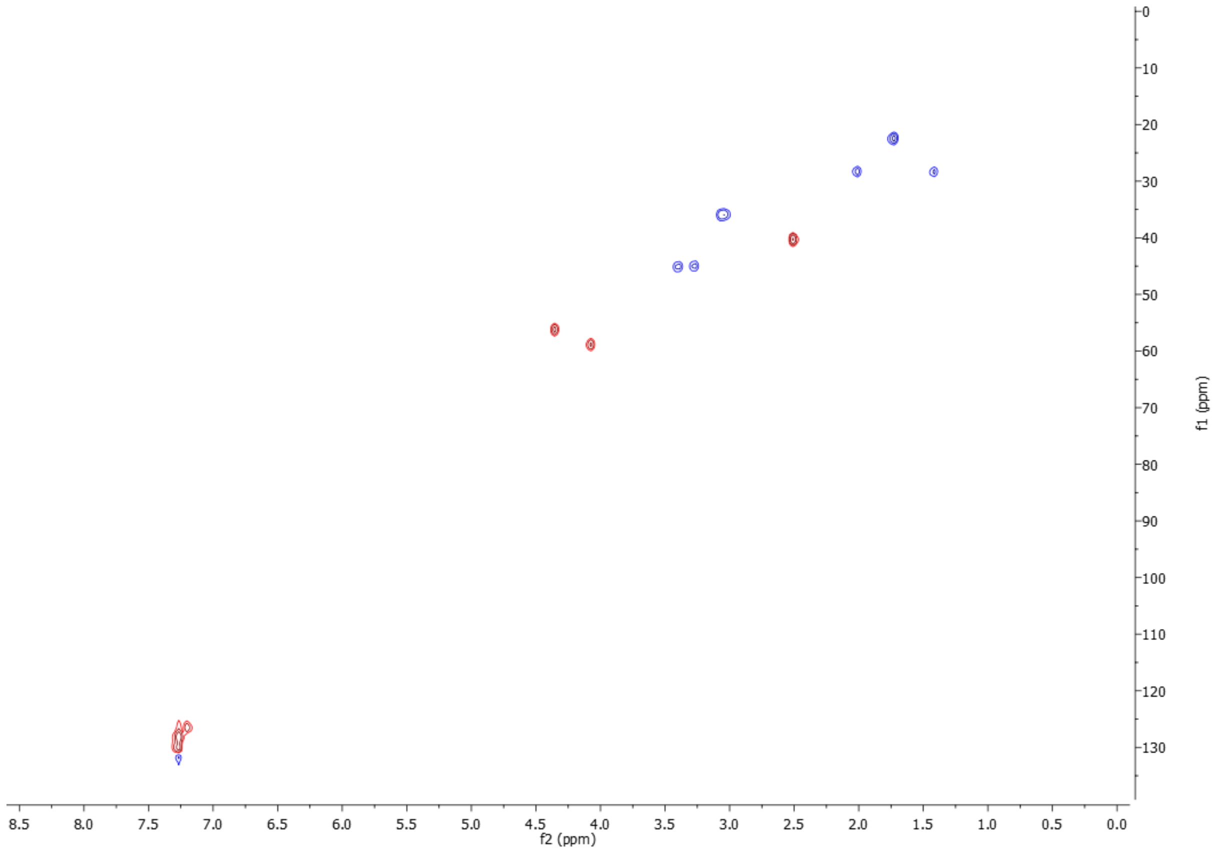

Figure S30 HMBC spectrum of compound 6 in DMSO

0

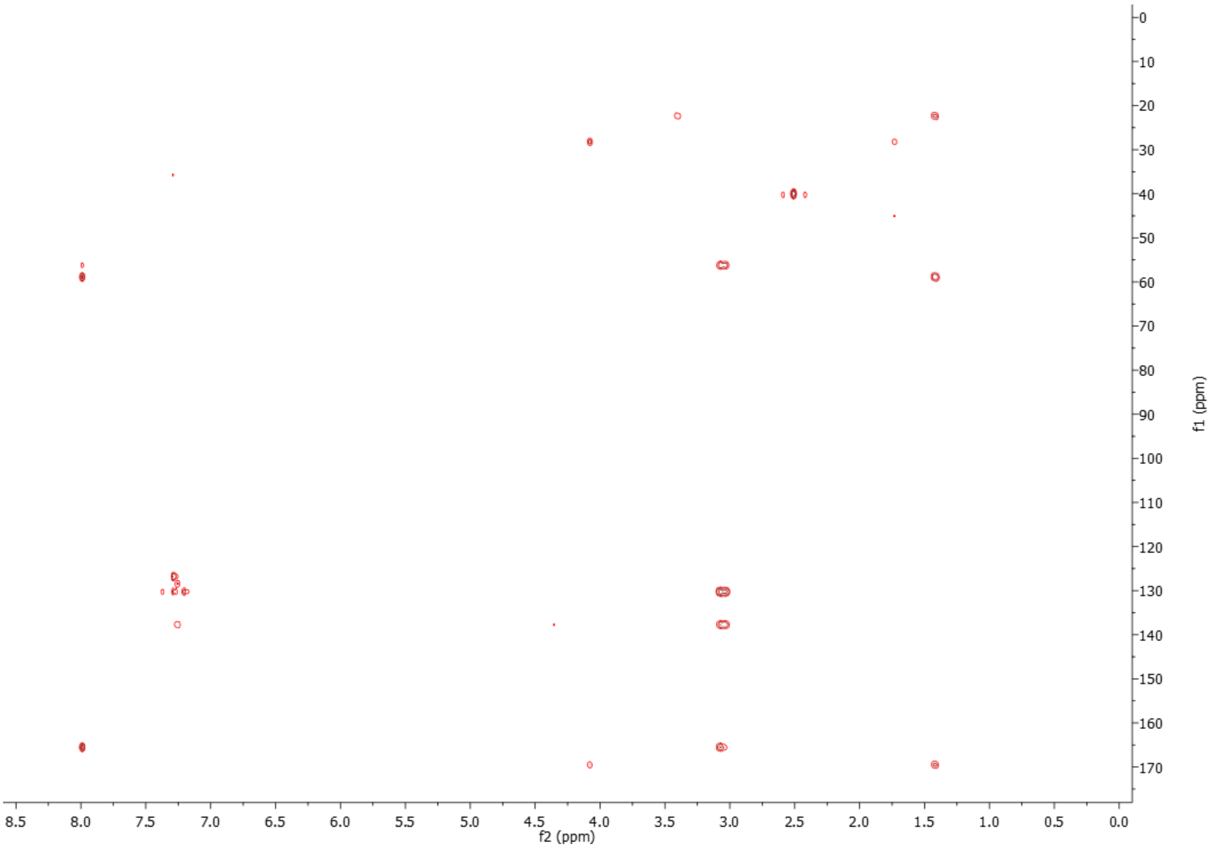

Table S7 Experimental NMR data of compound 7 in DMSO - d_6 at 25°C

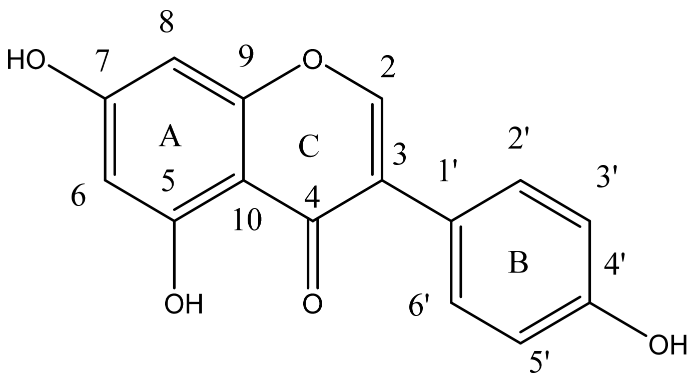

Part (ring)	Position	δ_C	δ_H (*J* in Hz)
C	2	154.8	8.32, s
C	3	121.3	-
C	4	180.1	-
A	5	162.6	12.99, s
A			6.25,
	6	99.9	*d*, 1.8J)
A	7	164.8	11.01
A	8	94.2	6.35,

			d, 1.8J
A, C	9	158.0	-
A, C	10	105.4	-
B	1’	122.0	-
B	2’	129.9	7.37, (doublet)
B	3’	114.9	6.82, (doublet)
B	4’	157.0	
B	5’	114.9	6.82, (doublet)
B	6’	129.9	7.37, (doublet)
	7-OH	-	11.01, s
	5-OH	-	12.99, s
	4’-OH		9.68, s

Figure S31 (+)-LRESIMS spectrum of compound 7

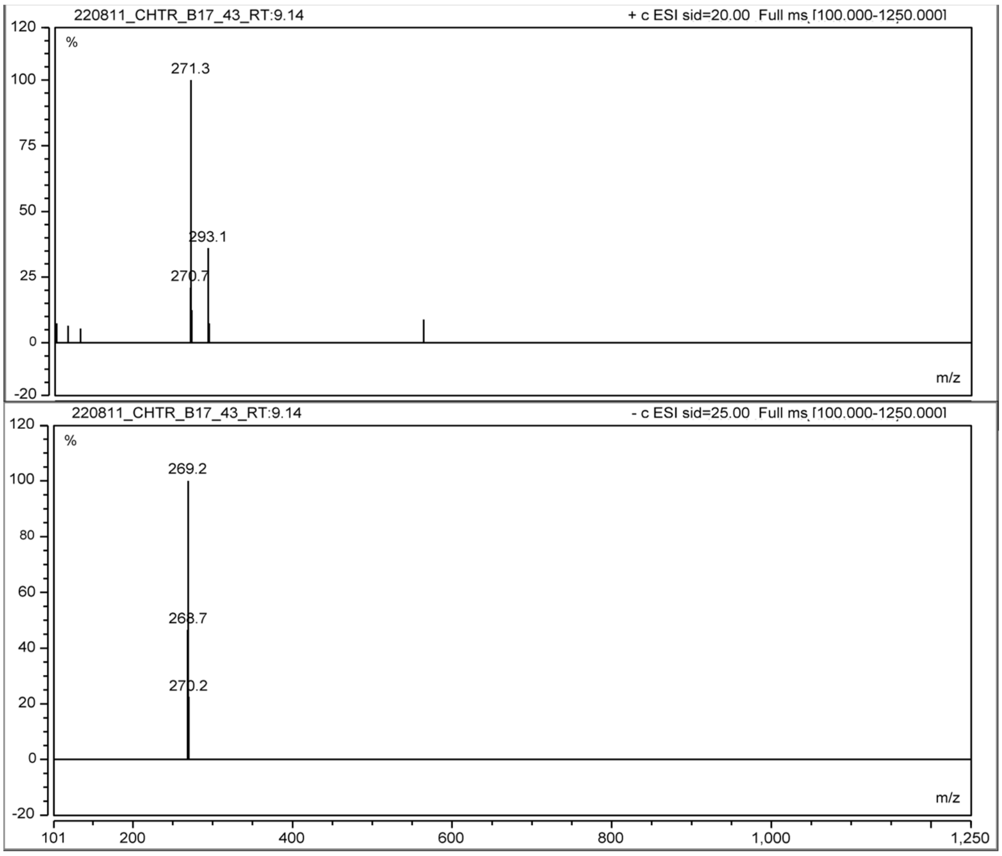

Figure S32 ^{1}H NMR (800 MHz, DMSO) spectrum of compound 7

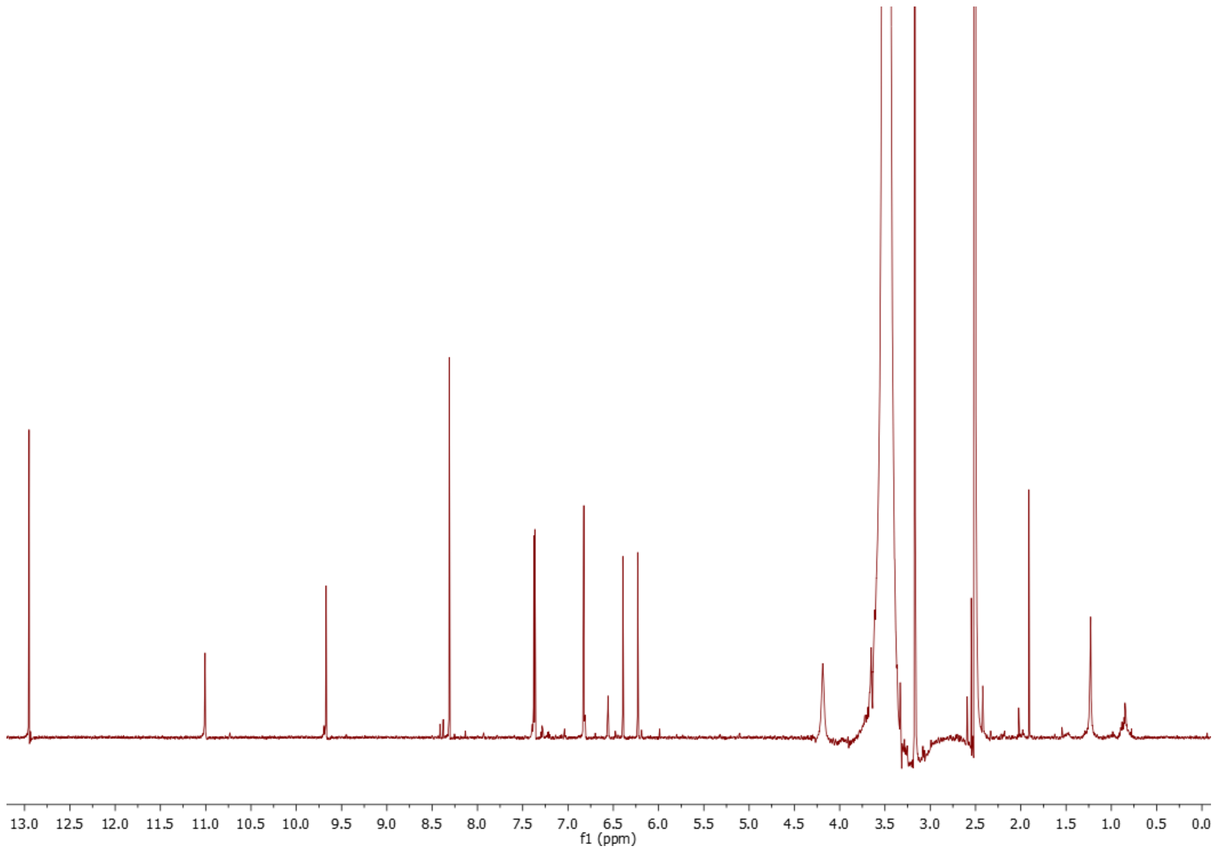

Figure S33 $^1H-^1H$ COSY spectrum of compound 7 in DMSO

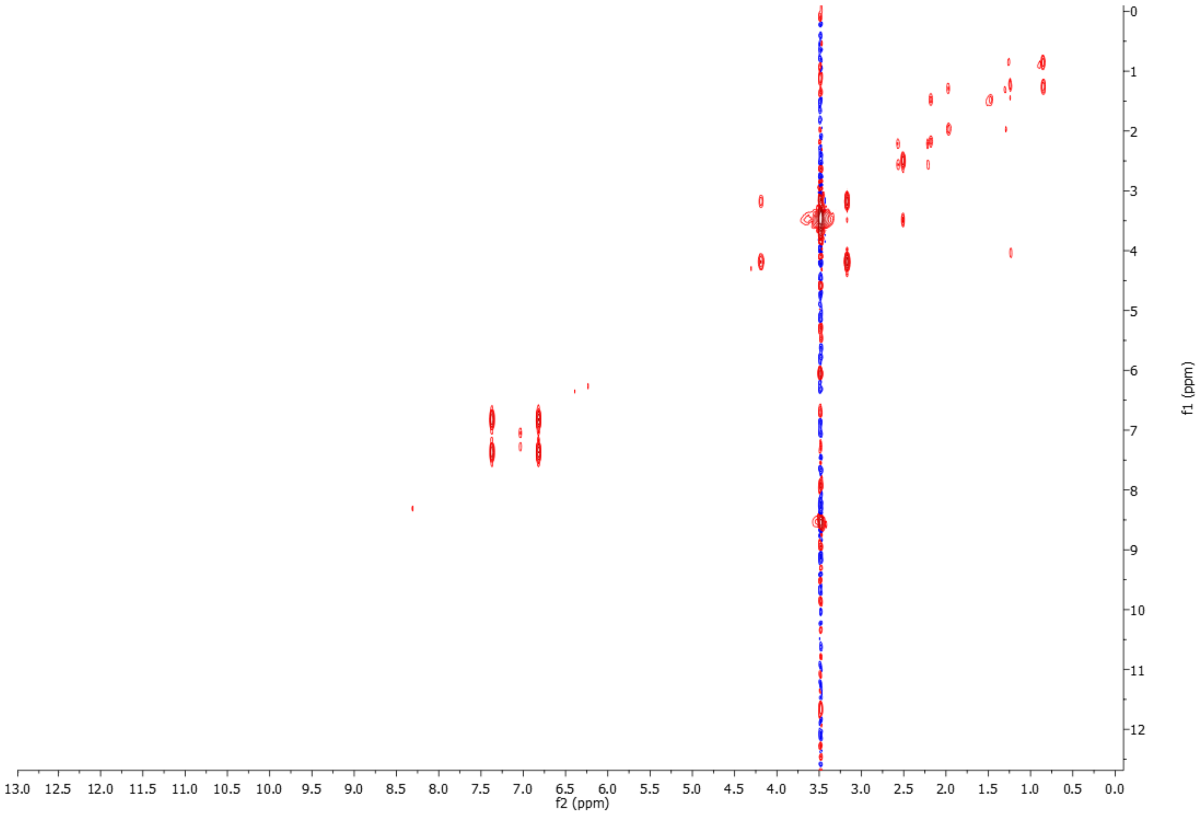

Figure S34 HSQC spectrum of compound 7 in DMSO

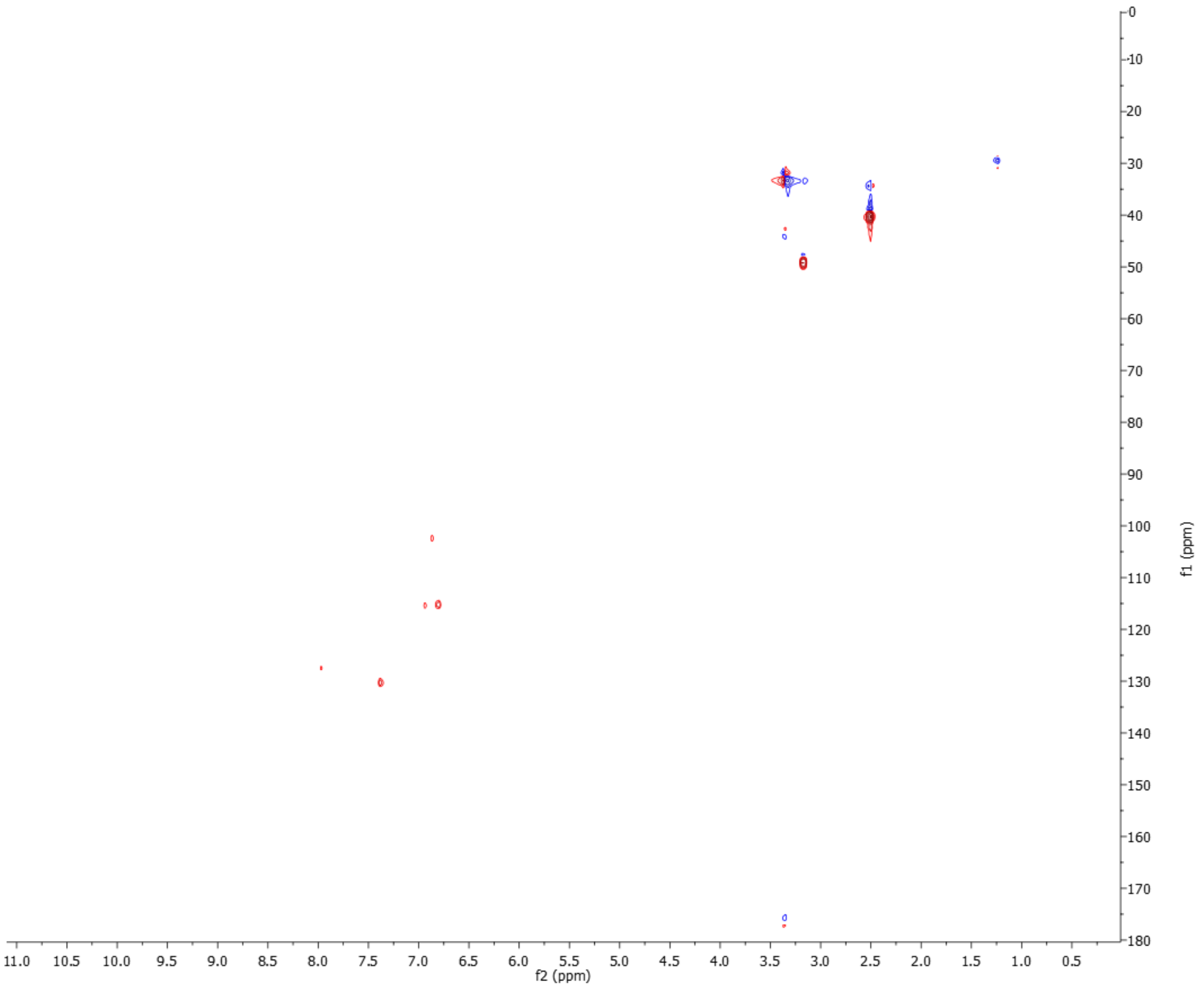

Figure S35 HMBC spectrum of compound 7 in DMSO

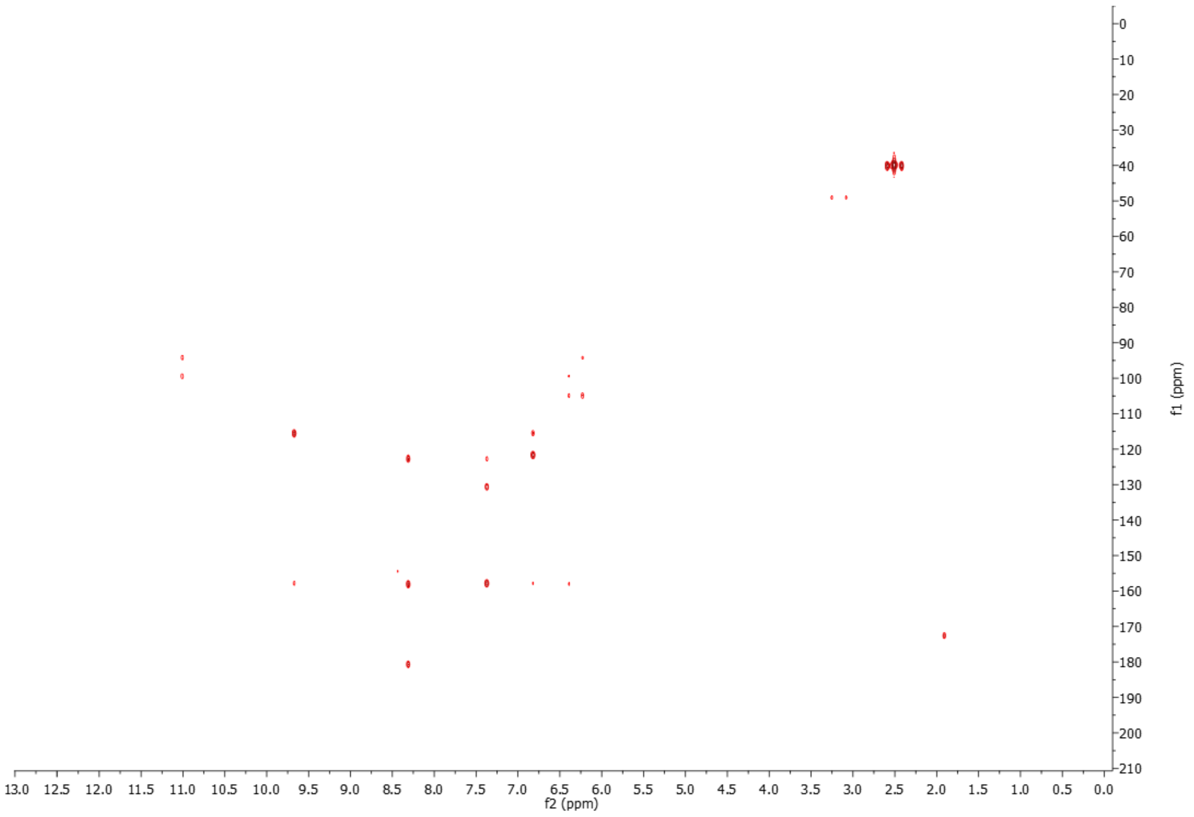

Table S8 Experimental NMR data of compound 8 in DMSO - d_6 at 25°C

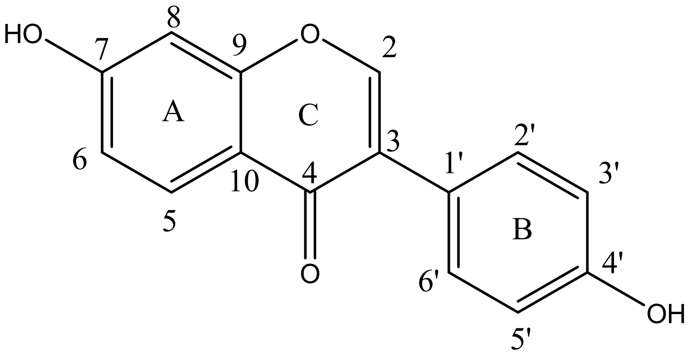

Part (ring)	Position	δ_C	δ_H (J in Hz)
C	2		
C	3	123.9	-
C	4	175.6	-
A	5	128.8	7.94, d, 7.9J
A	6	115.3	6.94, dd, 2.27J, 8.88J
A	7	163.3	-
A	8	102.6	6.82, *d*, 3.28*J*
A, C	9	157.7	-
A, C	10	117.3	-
B	1’	123.9	-
B	2’	130.8	7.38, *d*, 8.8*J*
B	3’	115.6	6.80, *d*, 8.8*J*
B	4’	157.7	-

B	5’	115.6	6.80, *d*, 8.8*J*
B	6’	130.8	7.38, *d*, 8.8*J*
	7-OH	-	10.76, *s*
	4’-OH	-	9.53, *s*

Figure S36 (+)-LRESIMS spectrum of compound 8

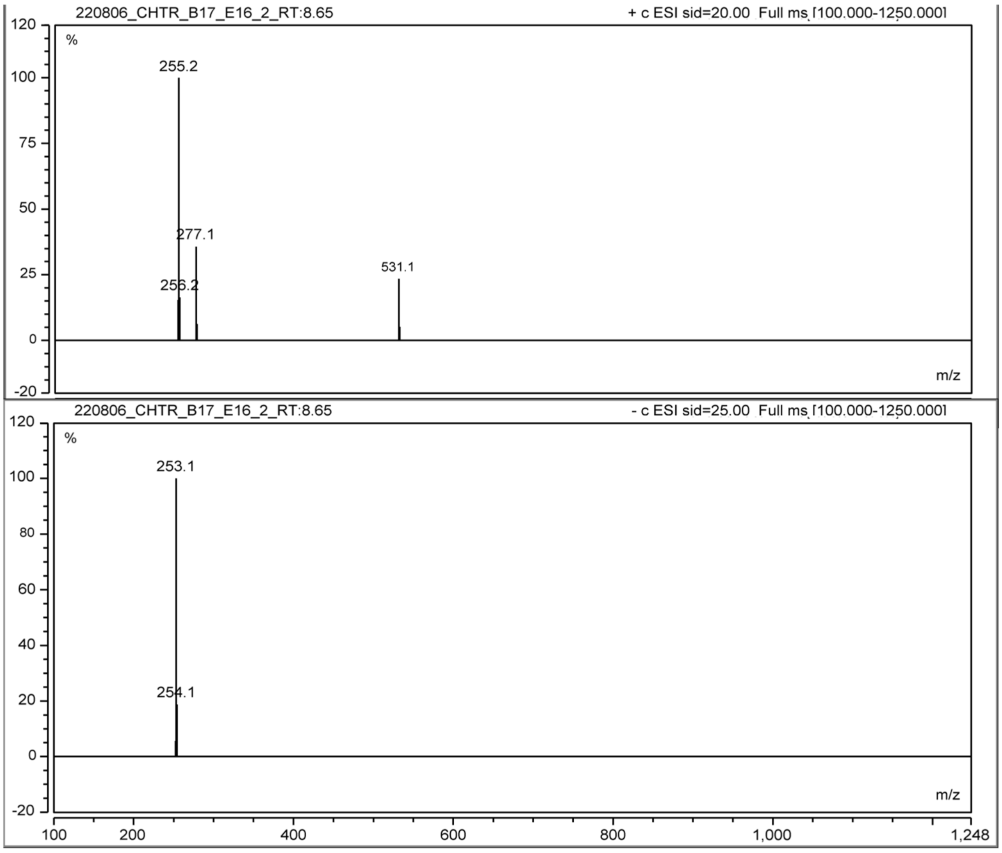

Figure S37 ^{1}H NMR (800 MHz, DMSO) spectrum of compound 8

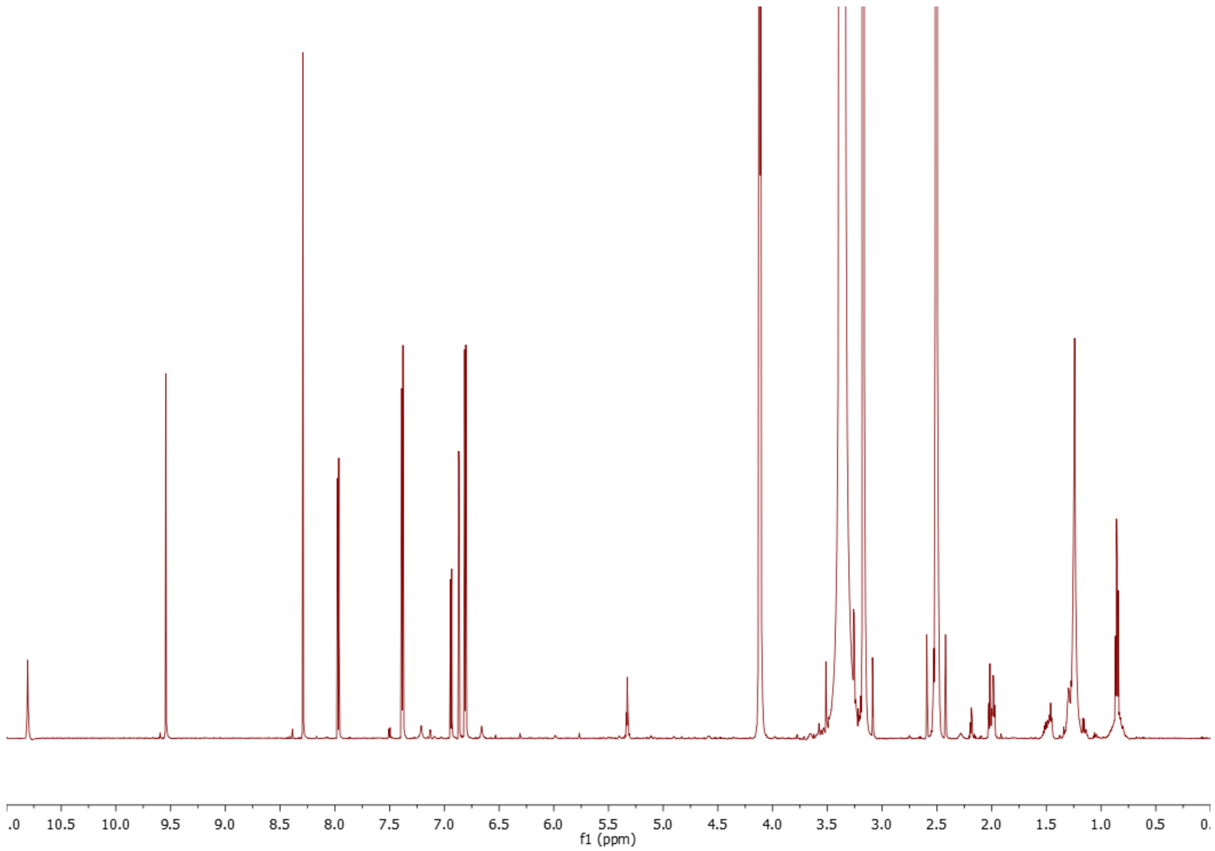

Figure S38 $^{1}H-^{1}H$ COSY spectrum of compound 8 in DMSO

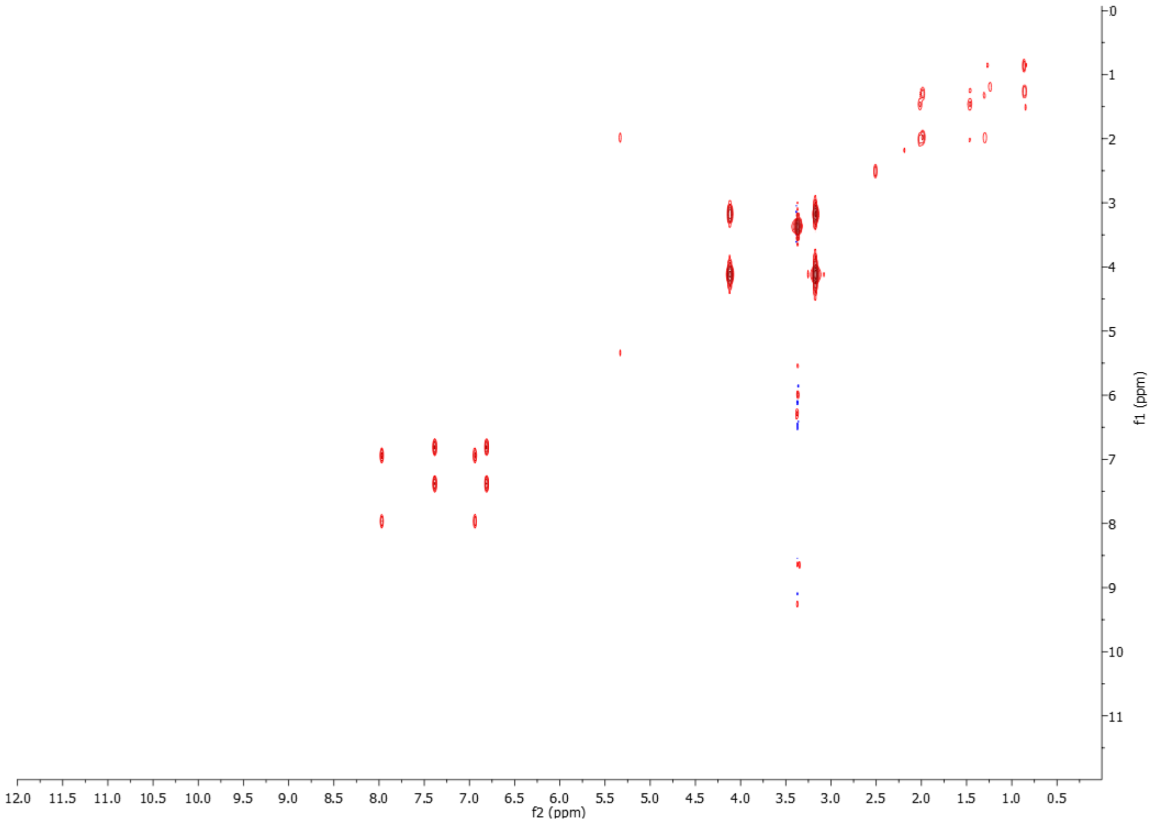

Figure S39 HSQC spectrum of compound 8 in DMSO

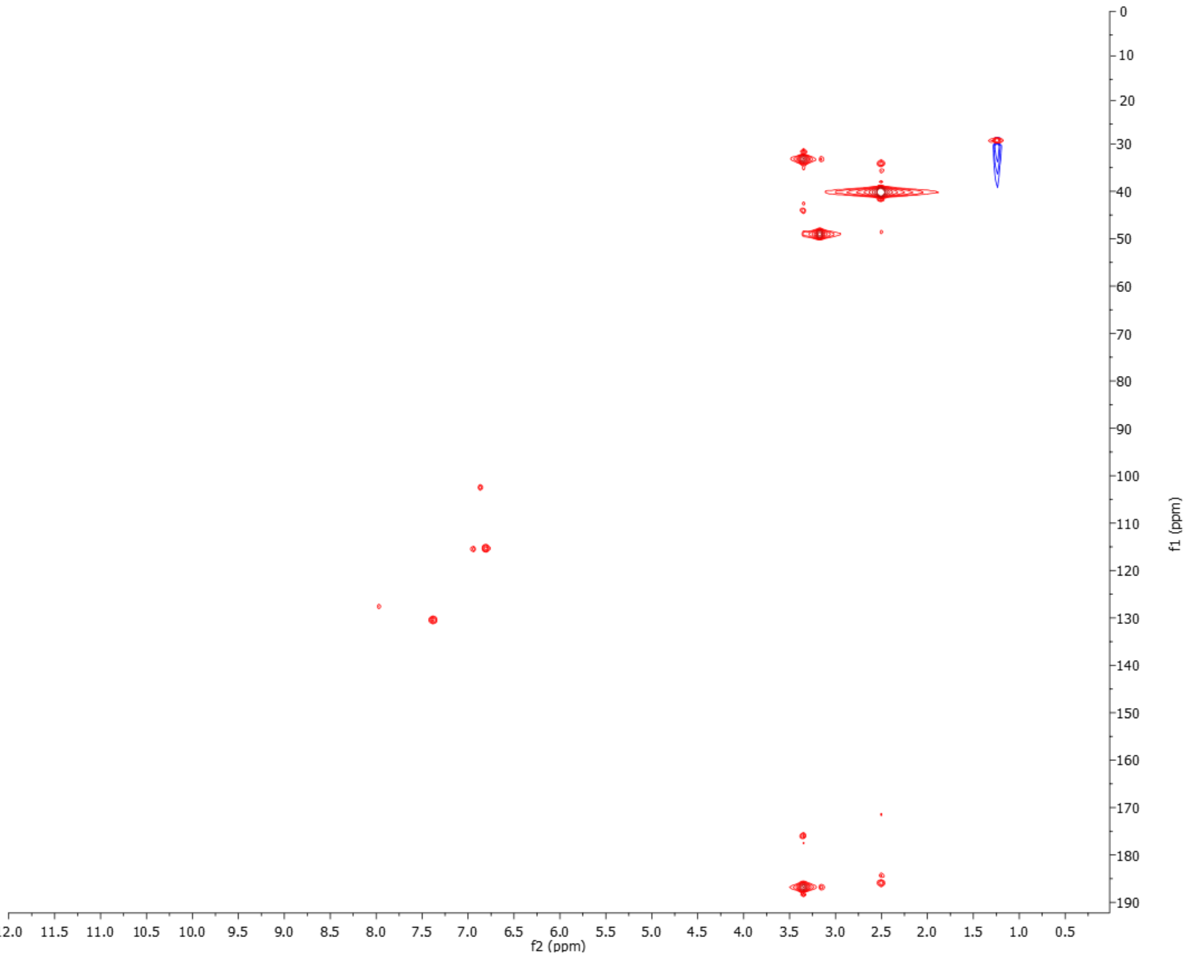

Figure S40 HMBC spectrum of compound 8 in DMSO

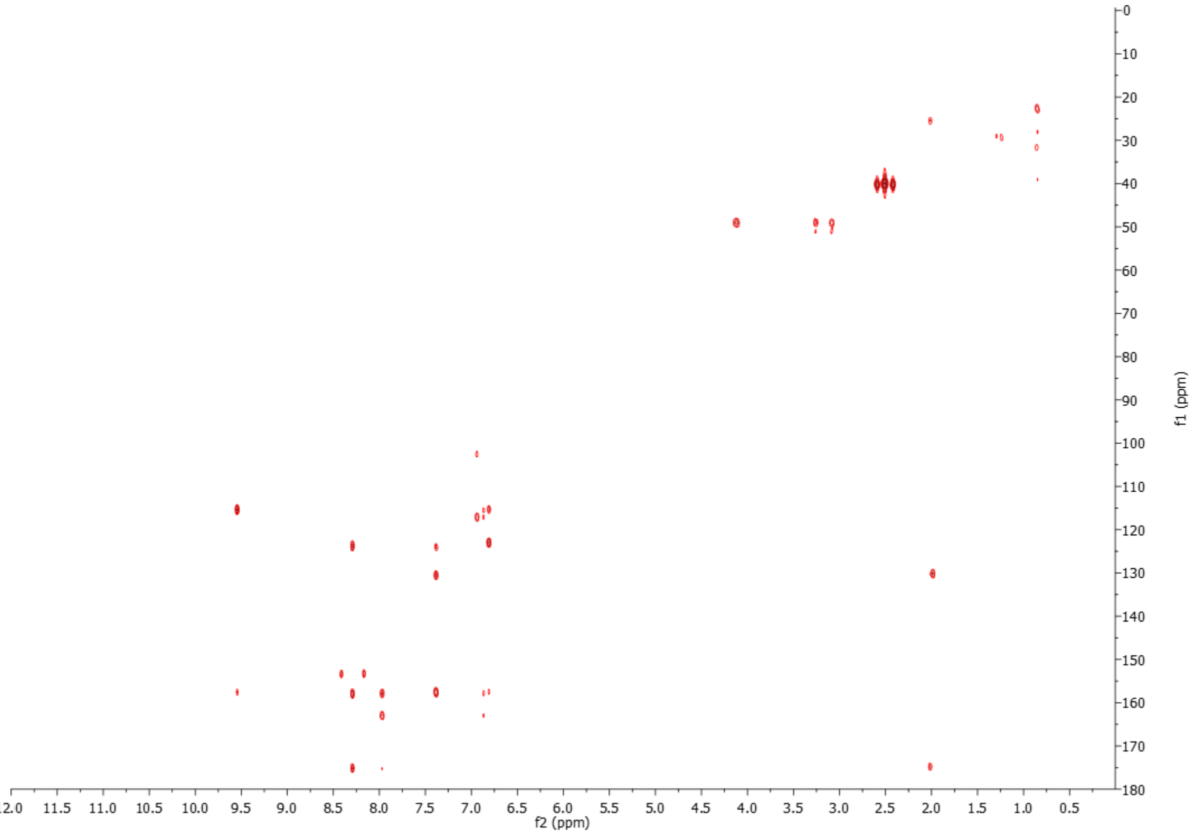

Figure S41 LC-MS chromatograms of caecum extracts produced from a single animal sample fed with *Bacillus* composition F1.

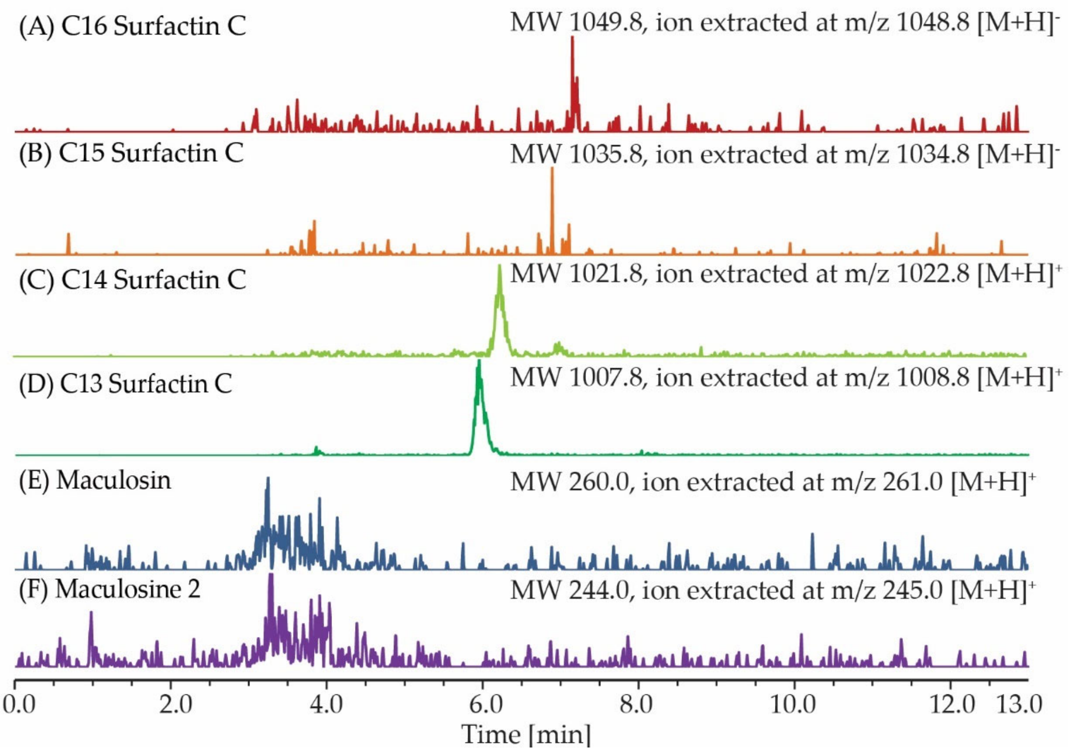

Table S9 Antimicrobial activity of EtoAc and Crude extracts of *Bacillus* strains

EtoAc Extract

Strain	200µg/mL					100µg/mL					50µg/mL					25µg/mL				
	C.P	E.C	P.A	S.A	S.E	C.P	E.C	P.A	S.A	S.E	C.P	E.C	P.A	S.A	S.E	C.P	E.C	P.A	S.A	S.E
BPR-17	✓	✓	✓	✓	✓	✓	✓	✓	✓	✓	✓	✓	✗	✓	✗	✓	✓	✗	✓	✗
BPR-16	✓	✓	✓	✓	✓	✓	✓	✓	✓	✓	✓	✓	✗	✓	✗	✓	✓	✗	✓	✗
BPR-14	✓	✓	✗	✓	✗	✓	✓	✗	✓	✗	✓	✓	✗	✓	✗	✓	✓	✗	✓	✗
BPR-13	✗	✗	✗	✗	✗	✗	✗	✗	✗	✗	✗	✗	✗	✗	✗	✗	✗	✗	✗	✗
BPR-12	✗	✗	✗	✗	✗	✗	✗	✗	✗	✗	✗	✗	✗	✗	✗	✗	✗	✗	✗	✗
BPR-11	✓	✓	✗	✓	✗	✓	✓	✗	✓	✗	✓	✓	✗	✓	✗	✓	✓	✗	✓	✗

Crude Extract

Strain	800µg/mL					400µg/mL					200µg/mL					100µg/mL				
	C.P	E.C	P.A	S.A	S.E	C.P	E.C	P.A	S.A	S.E	C.P	E.C	P.A	S.A	S.E	C.P	E.C	P.A	S.A	S.E
BPR-17	✗	✗	✗	✗	✗	✗	✗	✗	✗	✗	✗	✗	✗	✗	✗	✗	✗	✗	✗	✗
BPR-16	✗	✗	✗	✗	✗	✗	✗	✗	✗	✗	✗	✗	✗	✗	✗	✗	✗	✗	✗	✗
BPR-14	✗	✗	✗	✗	✗	✗	✗	✗	✗	✗	✗	✗	✗	✗	✗	✗	✗	✗	✗	✗
BPR-13	✗	✗	✗	✗	✗	✗	✗	✗	✗	✗	✗	✗	✗	✗	✗	✗	✗	✗	✗	✗
BPR-12	✗	✗	✗	✗	✗	✗	✗	✗	✗	✗	✗	✗	✗	✗	✗	✗	✗	✗	✗	✗
BPR-11	✗	✗	✗	✗	✗	✗	✗	✗	✗	✗	✗	✗	✗	✗	✗	✗	✗	✗	✗	✗

Figure S42 Stacked ^{1}H NMR spectra of *Bacillus* EtoAC extracts

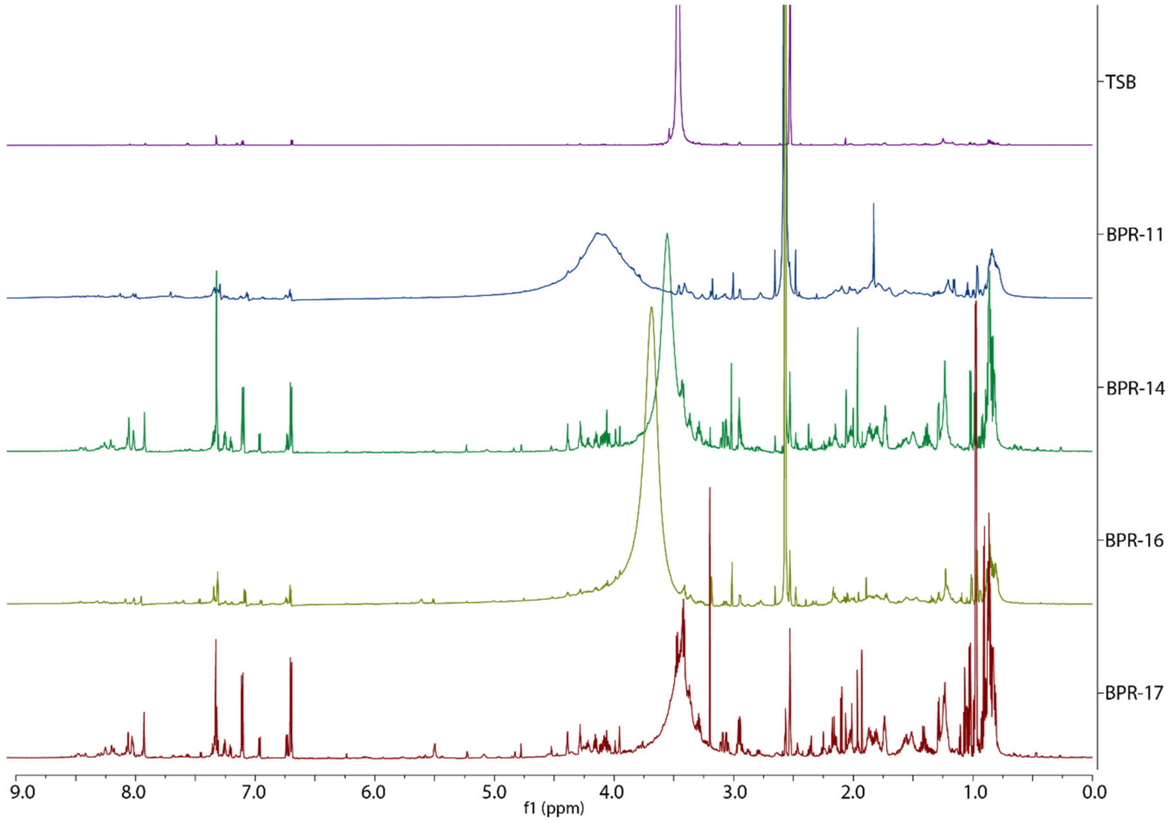

Supplement: Supplementary file 1 [file antibiotics-12-00407-s001.zip › antibiotics-2217155-supplementary.pdf]
